# Slow running benefits: Boosts in mood and facilitation of prefrontal cortex function even at very light intensity

**DOI:** 10.1162/IMAG.a.1297

**Published:** 2026-08-04

**Authors:** Chorphaka Damrongthai, Ryuta Kuwamizu, Yudai Yamazaki, Naoki Aoike, Dongmin Lee, Kyeongho Byun, Ferenc Torma, Kazuki Hyodo, Worachat Churdchomjan, Michael A. Yassa, Kazutaka Adachi, Hideaki Soya

**Affiliations:** Laboratory of Exercise Biochemistry and Neuroendocrinology, Institute of Health and Sport Sciences, University of Tsukuba, Ibaraki, Japan; Advanced Research Initiative for Human High Performance (ARIHHP), Institute of Health and Sport Sciences, University of Tsukuba, Ibaraki, Japan; Faculty of Physical Therapy and Sports Medicine, Rangsit University, Pathum Thani, Thailand; Department of Health and Sports, Niigata University of Health and Welfare, Niigata, Japan; Institute for Human Movement and Medical Sciences, Niigata University of Health and Welfare, Niigata, Japan; Division of Sport Science, Sport Science Institute and Health Promotion Center, College of Arts and Physical Education, Incheon National University, Incheon, Republic of Korea; Research Center for Molecular Exercise Science, Hungarian University of Sports Science, Budapest, Hungary; Physical Fitness Research Institute, Meiji Yasuda Life Foundation of Health and Welfare, Tokyo, Japan; Department of Neurobiology and Behavior, University of California, Irvine, CA, United States; Center for the Neurobiology of Learning and Memory, University of California, Irvine, CA, United States; Department of Health Sciences, SBC Tokyo Medical University, Chiba, Japan; Center for Cybernics Research, University of Tsukuba, Ibaraki, Japan

**Keywords:** locomotion, executive function, fNIRS, mild exercise, pleasant mood, prefrontal cortex

## Abstract

Although running upright has been reported to have positive effects on both physical and mental health, the minimum running intensity/speed that would benefit mood and prefrontal cognition is not yet clear. Here, we tested the hypothesis that a brief bout of very slow running, which is classified as a very light intensity exercise, would enhance positive mood and executive function and increase activation in executive-function–related prefrontal subregions. Twenty-four healthy participants completed a 10-minute very slow running session on a treadmill at 35% *V̇*o_2peak_ and a resting control session in randomized order. Executive function was measured using the Stroop task and mood state was measured using the Two-Dimensional Mood Scale (TDMS) before and after both sessions. Cortical hemodynamic changes while performing the task were monitored using functional near-infrared spectroscopy (fNIRS). The results show that 10 minutes of very slow running significantly enhanced mood, reduced Stroop interference time (i.e., enhanced executive function), and elicited left lateral PFC activation. Moreover, head acceleration, the magnitude of up-and-down oscillations, was measured during running, and a significant positive correlation with pleasant mood was found. Head acceleration is a remarkable characteristic of running and may be one of the factors related to a pleasant mood induced by very slow running. In conclusion, the current study reveals that a single bout of running, even at very slow speed, elicits a pleasant mood and improved executive function with enhancing activation in prefrontal subregions. This sheds light on the slow running benefits to brain health.

## 1 Introduction

Running has an extremely long history as a means of locomotion for humans. Human beings can navigate long distances through bipedal running (Bramble & Lieberman, 2004). It is interesting to hypothesize that human upright bipedal running is also strongly tied to the evolutionary expansion of the Homo sapiens’ frontal lobes and higher cognitive functions (Schulkin, 2016). Against this background, running may be viewed not simply as another exercise modality, but as a biologically foundational form of human movement with particular relevance to brain function.

Running not only decreases the risk of cardiovascular diseases in general but also has positive effects on mental and brain health. There are numerous studies reporting the effects of acute exercise on cognitive function (Pontifex et al., 2019), but major studies often focus on cycling, with which exercise intensity is easier to control. Accordingly, although acute exercise is known to benefit cognition, it remains unclear whether running produces comparable effects on executive function and prefrontal cortical activity, particularly at very light intensity. There is a limited number of studies that have focused on running with precisely defined intensities; among these, research exploring neural mechanisms has predominantly used event-related potentials derived from electroencephalographic data (Hillman et al., 2003; Hung et al., 2016; McGowan et al., 2019; Shigeta et al., 2021; Themanson & Hillman, 2006; Wohlwend et al., 2017). Consequently, few past studies have used neuroimaging techniques that allow region-specific analyses to examine the effects of acute running on cognitive function in subregions of the prefrontal cortex (PFC). Additionally, the few existing studies that used functional near-infrared spectroscopy (fNIRS) did not perform region-specific analyses within the PFC (Lambrick et al., 2016; Takahashi & Grove, 2023). Thus, a key unresolved issue is whether a single bout of running facilitates executive function together with activation of anatomically defined prefrontal subregions.

Of particular interest is the dorsolateral PFC (DLPFC), which has long been considered a brain region crucial for executive function (Cools & Arnsten, 2022; Frings et al., 2018; MacDonald et al., 2000). One of our previous studies used multichannel fNIRS to show that moderate-intensity running exercise (50% *V̇*o_2peak_) activates the DLPFC and frontopolar areas (FPA) of both hemispheres and enhances executive function (Damrongthai et al., 2021), extending previous pedaling findings (Byun et al., 2014b; Kujach et al., 2018; Kuwamizu et al., 2023; Yanagisawa et al., 2010). However, moderate-intensity exercise is the intensity at which the hypothalamic stress response occurs (Soya et al., 2007). As such, vulnerable individuals, such as those with low fitness levels and older adults, may find it difficult to adhere to moderate-intensity running, which is relatively fast and imposes greater joint loading than pedaling exercise. It is, therefore, important to determine whether comparable effects can be elicited at a lower, stress-response-free intensity and at an extremely slow speed, close to normal walking speed (about 4–6 km/h). If so, the cognitive and neural benefits of running would not be limited to more demanding exercise intensities.

Activation in the PFC can be measured using fNIRS, a non-invasive brain imaging instrument suitable for treadmill-based paradigms. In the last two decades, fNIRS has contributed to research on brain activity during locomotion, including walking (Miyai et al., 2001; Vitorio et al., 2017). Importantly, however, motion artifacts and skin blood flow may contaminate fNIRS signals measured during locomotion (Miyazawa et al., 2013; Vitorio et al., 2017), making it challenging to isolate cortical activity. To address this problem, one of our previous studies focused on executive function after treadmill running (Damrongthai et al., 2021). We developed an experimental protocol to minimize non-cortical fNIRS signals by examining the decay of relevant physiological indicators after exercise (Byun et al., 2014a, 2014b; Damrongthai et al., 2021; Kujach et al., 2018; Yanagisawa et al., 2010). We have also examined exercise-related changes in prefrontal subregions engaged during the color-word Stroop task (CWST), a representative task of inhibitory control, using the virtual registration method (Damrongthai et al., 2021; Tsuzuki & Dan, 2014; Tsuzuki et al., 2007; Yanagisawa et al., 2010). This approach enables anatomically standardized evaluation of prefrontal subregions across participants. This, in turn, allows us to test, within a controlled laboratory treadmill paradigm, whether very slow running improves executive function and increases prefrontal activation.

Regarding affect, our previous study showed a notable increase in positive mood with moderate-intensity (50% *V̇*o_2peak_) running (Damrongthai et al., 2021). However, intensities around or above the ventilatory threshold may sometimes hinder this positive mood and induce unpleasant feelings (Ekkekakis et al., 2008). If both mood and executive function improve with very slow running, very light-intensity running may represent an exercise mode that benefits affect and cognition while minimizing stress, fatigue, and discomfort. Even very slow running may enhance arousal by stimulating the reticular activating system, which regulates ascending projections to the PFC, a region important for both cognition and mood regulation (Basso & Suzuki, 2017; Hiraga et al., 2024; Kuwamizu et al., 2025). Therefore, in this study, we assessed mood using the Two-Dimensional Mood Scale (TDMS) (Sakairi et al., 2013), which expresses arousal and pleasantness on two axes, allowing us to determine whether a single bout of very slow running confers simultaneous benefits to mood and executive function.

Running may also have characteristics distinct from other exercise modes that are relevant to its neural or affective effects. Compared with walking, running involves greater rhythmic impact and larger vertical oscillations of the body. Experimental findings in rodents suggest that vertical acceleration may act as a mechanical stimulus to the brain (Ryu et al., 2020). Thus, the characteristic vertical oscillations of running may represent one candidate factor underlying the mood- or cognition-related benefits of running. To explore this possibility, we measured head acceleration (HA) during running. Because HA remains underexplored and its relevance to exercise-induced mood and cognitive benefits has not been established, this analysis was treated as exploratory.

Accordingly, this study tested whether a single bout of very slow running at very light intensity improves mood and executive function, and whether such improvements are accompanied by facilitation of activity in prefrontal subregions measured by fNIRS. In addition, associations between head acceleration and behavioral or neural outcomes were explored as a potential mechanistic clue.

## 2 Methods

### 2.1 Participants

Participants included 24 young adults who were Japanese native speakers. All of them were nonsmokers, were right-hand dominant, had normal or corrected-to-normal vision, and had normal color vision. No participant reported a history of neurological or psychiatric disorders or had any conditions requiring medical care. One additional participant was tested but was excluded from the final analyses because her behavioral data change (Stroop interference reaction-time difference during her participation in the running condition) fell outside the range of 3 standard deviations from the mean. The demographic data of the participants are shown in Table 1. The study was approved by the Institutional Ethics Committee of the University of Tsukuba (approval number: tai020-120) where the protocol corresponded to the latest version of the Declaration of Helsinki. Written informed consent was obtained from all participants.

**Table 1.** Participant demographics data (n = 24).

	Male (*n* = 13)	Female (*n* = 11)
Age (years)	23.38 ± 1.76	20.55 ± 2.07
Weight (kg)	65.31 ± 11.65	49.12 ± 4.05
Height (cm)	172.15 ± 5.34	159.06 ± 4.03
BMI (kg/m^2^)	22.03 ± 3.92	19.40 ± 1.39
Graded exercise test		
*V̇*o_2peak_ (mL/kg/min)	48.24 ± 6.25	40.03 ± 3.84
HR_max_ (bpm)	191.15 ± 8.43	190.00 ± 7.25
R	1.14 ± 0.03	1.13 ± 0.06
RPE	19.23 ± 0.60	19.27 ± 1.01
Final treadmill speed (km/h)	16.54 ± 2.40	13.55 ± 1.97

Presented values are mean ± SD.

BMI = Body Mass Index; *V̇*o_2peak_ = Peak oxygen uptake; HR_max_ = Maximal Heart Rate; R = Respiratory exchange ratio; RPE = Rate of Perceived Exertion.

### 2.2 Procedure for main experiment

**Fig. 1.**
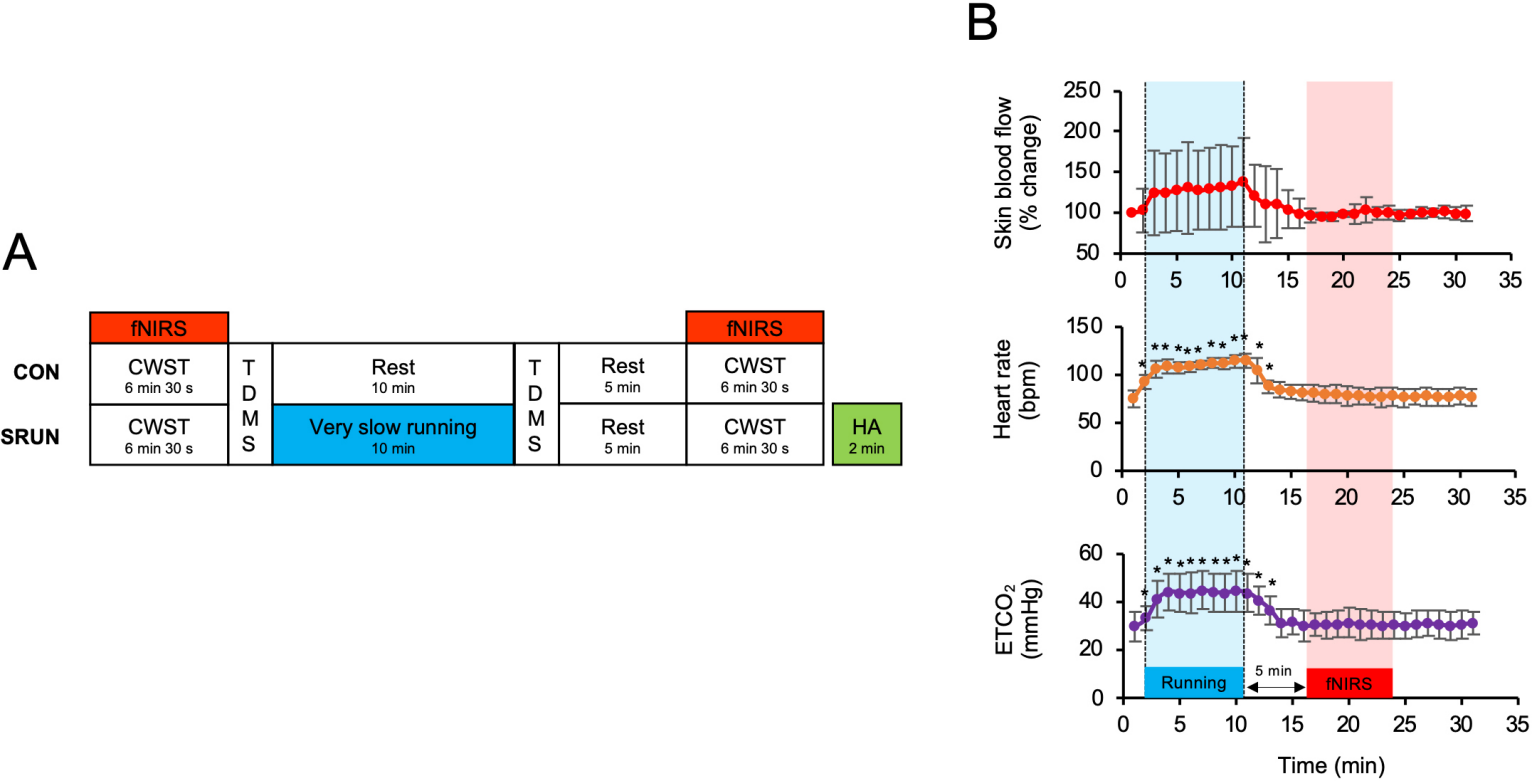
(A) Experimental design for investigating the acute effect of very slow running on prefrontal cortical executive function. CON = control session, SRUN = very slow running session, CWST = color-word Stroop task, TDMS = Two-Dimensional Mood Scale, fNIRS = Functional near-infrared spectroscopy, HA = Head acceleration. (B) Preliminary results of the non-cortical physiological parameters at baseline, during 10 minutes of very slow running and 20 minutes rest. Time points with significant change compared to baseline are shown with asterisks (*p* < 0.05, one-way ANOVA with Dunnett correction). All variables returned and stabilized at baseline levels within 5 minutes. Thus, fNIRS measurement was started 5 minutes after running to eliminate possible non-cortical signal contamination. bpm = beats per minute, ETCO_2_ = End-tidal carbon dioxide.

The main experiment consisted of two sessions conducted on separate days: control (CON) and very slow running (SRUN) (Fig. 1). During the CON session, participants were instructed to sit on a chair placed on a stationary treadmill for 10 minutes without engaging in any physical activity. Following our previous study (Damrongthai et al., 2021), a seated, rather than standing, resting condition was chosen to minimize physiological variability from postural changes and to ensure a stable resting state. This choice also reflects typical sedentary behavior in daily life, making it suitable for evaluating the effects of very slow running. In contrast, during the SRUN session, participants were asked to run at a very slow pace on the treadmill for 10 minutes. The order of the sessions was randomized and conducted using a counterbalanced design to minimize any potential order effects. Before the main experiment, the maximal oxygen uptake (*V̇*o_2peak_) of each participant was measured to appropriately personalize the very light exercise intensity, which is to say treadmill speed, for very slow running. For safety during the *V̇*o_2peak_ test, participants wore an overhead safety harness to prevent falls; the harness did not provide body-weight support and did not restrict natural running movement. The target was 35% *V̇*o_2peak_ in accordance with the American College of Sports Medicine (ACSM) guidelines (Pescatello et al., 2014) (Supplemental Material 1). We set the exercise duration to 10 minutes because our previous work has indicated that a minimum of 10 minutes of exercise enhances Stroop task performance and prefrontal activation (Yanagisawa et al., 2009). To verify whether the participants could perform very slow running or a very light-intensity exercise, we monitored heart rate (HR) and rate of perceived exertion (RPE) during exercise. Participants performed the CWST and completed mood assessments before and after each session (both resting and running). Prefrontal hemodynamic changes during the CWST were monitored using fNIRS. To avoid contamination from non-cortical physiological signals, such as skin blood flow, HR, and end-tidal carbon dioxide (ETCO_2_), fNIRS measurements were conducted 5 minutes after the SRUN session. Very slow running can induce non-cortical physiological signals (skin blood flow, HR, and ETCO_2_) that could interfere with the fNIRS signal, so taking measurements 5 minutes after very slow running was deemed appropriate because within that time all physiological variables completely return to baseline (Fig. 1B; Supplemental Material 2). HA during running was assessed after the SRUN session to eliminate any influence from the equipment worn on the head. By measuring HA on the same day, we quantified each participant’s HA. Mood state was assessed before and after very slow running using the TDMS.

### 2.3 Behavioral measurements

**Fig. 2.**
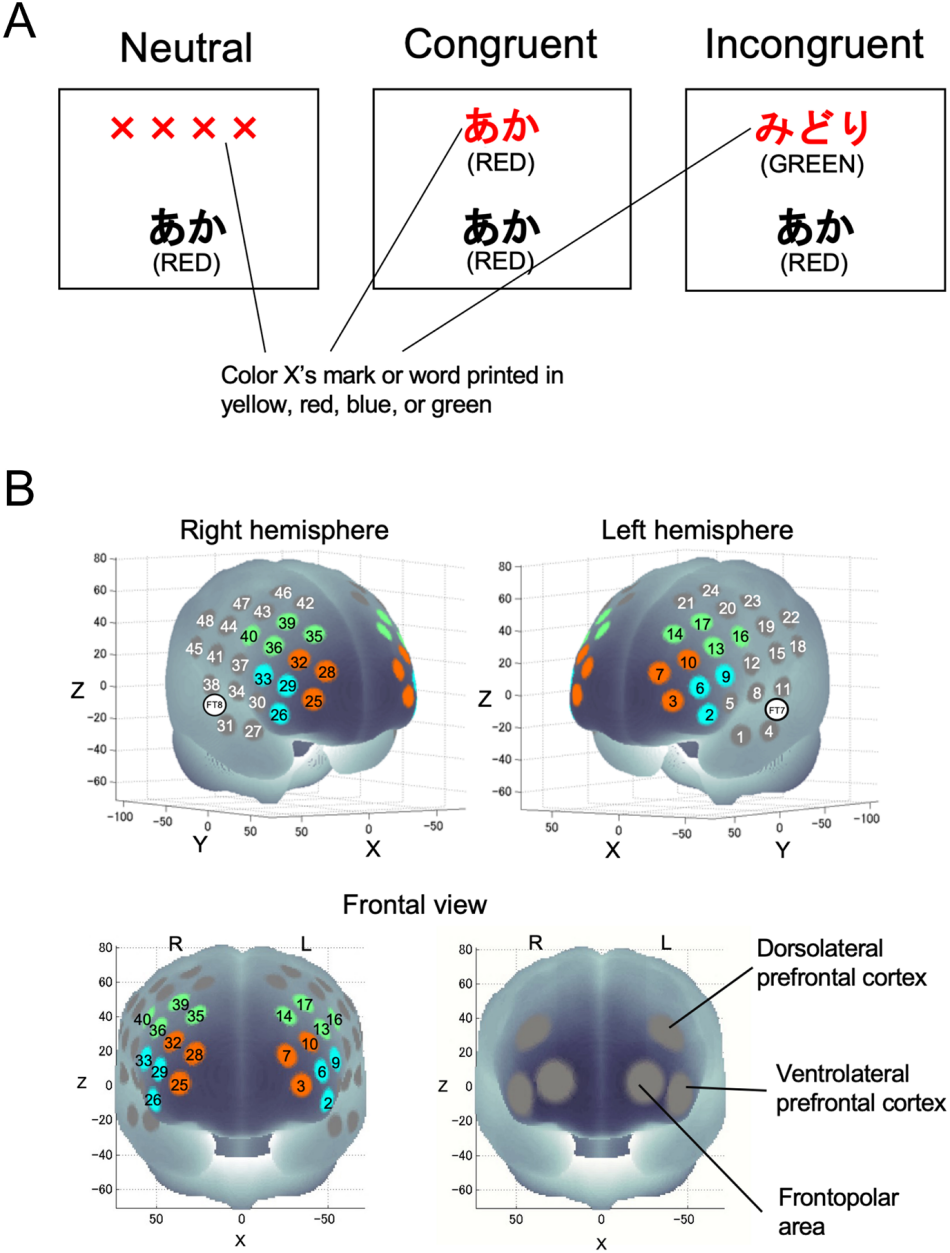
(A) Examples of the color-word Stroop test neutral, congruent, and incongruent conditions. The presented words were written in Japanese. English translations are shown in parentheses. (B) Spatial profiles of fNIRS channels used. Two sets of probe holders were placed to cover both lateral prefrontal activation foci as in our previous studies. Colors indicate each region of interest (ROI) in the lateral part of the PFC.

An event-related version of the CWST was used to evaluate executive function. This version of the CWST was developed for neuroimaging with functional magnetic resonance imaging (fMRI) and fNIRS, and has been used in many previous studies (Damrongthai et al., 2021; Schroeter et al., 2002; Yanagisawa et al., 2010; Zysset et al., 2001). The test was displayed on a monitor with two rows of letters. The participants were instructed to decide whether the color of the letters in upper row corresponded to the color name in the lower row. They were also asked to place their index fingers on “yes” and “no” buttons and to respond to the test by pressing the correct button as quickly as they could. Subsequently, reaction time (RT) and error rate (ER) were calculated. The CWST consisted of three conditions: neutral, congruent, and incongruent. For the neutral condition, the upper row displayed a row of X’s (XXXX) printed in red, green, blue, or yellow, and the lower row displayed the word ‘RED’, ‘GREEN’, ‘BLUE’, or ‘YELLOW’ printed in black. For the congruent condition, the upper row displayed the word ‘RED’, ‘GREEN’, ‘BLUE’, or ‘YELLOW’ printed in the congruent color (e.g., ‘BLUE’ was printed in blue), and the lower row displayed the same words as in the lower row of the neutral condition. For the incongruent condition, the upper row displayed a color word printed in an incongruent color to produce interference between the color word and the color name (e.g., ‘YELLOW’ was printed in red), and the lower row displayed the same words as in the lower row of the neutral and congruent conditions (Fig. 2A). Each experimental session consisted of 30 trials, made up of 10 neutral, 10 congruent, and 10 incongruent trials, which appeared in random order. The upper row was presented 100 ms before the lower row in order to shift visual attention. Each trial remained on the screen until the participant responded or for 2 seconds, whichever was shorter. Then, a fixation cross appeared on the screen as an inter-stimulus interval for 10–12 seconds to avoid prediction of the timing of the subsequent trial. The number of trials (30) and the task duration (6.5 minutes) were set to ensure participants could maintain adequate concentration throughout the task when measuring fNIRS using an event-related model. Stroop interference, an index of executive function in the PFC, was calculated as the difference in RT between the incongruent and neutral conditions. All words were written in Japanese. The participants performed three practice sessions to ensure that they understood and were familiarized with the CWST well before starting the experiment.

### 2.4 fNIRS measurements

Cortical hemodynamic changes were monitored using the multichannel fNIRS optical topography system ETG-7000 (Hitachi Medical Corporation, Japan) with two wavelengths of near-infrared light (785 and 830 nm). The optical data from fNIRS were analyzed in accordance with the modified Beer–Lambert Law, although a specific differential pathlength factor was not applied, as the effective optical pathlength is difficult to determine accurately in human tissue (Maki et al., 1995). Instead, we calculated relative changes in oxy-Hb concentrations and expressed them in arbitrary units of millimolar-millimeters (mM ∙ mm), following previous studies (e.g., Maki et al., 1995). Oxy-Hb was selected as the index of cortical activation because it provides a higher signal-to-noise ratio and a stronger correlation with the BOLD response than deoxy-Hb (Hoshi et al., 2001), and has been consistently adopted in the same fNIRS system (e.g., Yanagisawa et al., 2010). The sampling rate was set at 10 Hz. For fNIRS probe placement, two sets of 4 × 4 multichannel probe holders, which consist of 8 illuminative and 8 detective probes arranged alternately at an inter-probe distance of 3 cm resulting in 24 channels (CH) per set, were placed to cover both lateral prefrontal activation foci as in previous studies. The left probe holder was placed such that probe 5 (between CH4 and CH11) was placed over FT7, with the medial edge of the probe column parallel to the medial line. The right probe holder was symmetrically placed on the left hemisphere (Fig. 2B). We adopted a virtual registration method to register fNIRS data to Montreal Neurological Institute (MNI) standard brain space (Tsuzuki et al., 2007), following previous studies (Byun et al., 2014a, 2014b; Damrongthai et al., 2021; Kujach et al., 2018; Yanagisawa et al., 2010). With this method, we were able to place a virtual probe holder on the scalp by simulating the holder’s deformation and by registering probes and channels onto a reference brain in the MRI database (Brett et al., 2002; Tsuzuki & Dan, 2014). We probabilistically estimated the MNI coordinate values for fNIRS in order to obtain the most likely microanatomical predictions for locations of the given channels as well as the spatial variability of the estimation (Singh et al., 2005). Finally, a MATLAB function was used to label the estimated locations in the Montreal Neurological Institute (MNI) standard space macro-anatomical brain atlas and coordinate system (Shattuck et al., 2008).

### 2.5 Analysis of fNIRS data

Prefrontal oxyhemoglobin (oxy-Hb) changes that occurred while performing the CWST were calculated as shown in our previous studies. Individual timeline data for each channel were preprocessed with a band-pass filter using a high-pass filter (0.04 Hz) to remove baseline drift and a low-pass filter (0.30 Hz) to screen out high-frequency noise (such as instrumental noise and heartbeat pulsations). The motion artifacts were checked by using the optical topography analysis tools (POTATo) (Hitachi, Ltd., Japan) (Sutoko et al., 2016). First, channels with poor probe contact and characterized by peak-to-peak constant fluctuations on the order of ±1 mM ∙ mm or more (0.6% of all channels) were excluded. Additionally, trials exhibiting synchronized fluctuations across multiple probes (Sutoko et al., 2016) or large deoxyhemoglobin (deoxy-Hb) fluctuations, particularly those showing a positive correlation change with oxy-Hb (Cui et al., 2010), with amplitudes approaching ±0.3 mM ∙ mm (Byun et al., 2014b) were inspected. Then, channel-wise and subject-wise contrasts were calculated by the inter-trial mean of differences between peak (4–11 seconds after trial onset) and baseline (0–2 seconds before trial onset) periods (Byun et al., 2014b; Damrongthai et al., 2021; Kuwamizu et al., 2021). The contrasts obtained were subsequently subjected to a second level of group analysis. This study adopted LBPA40, a widely used method among anatomical labeling systems, to combine 3 to 4 neighboring channels to form each region of interest (ROI) (Shattuck et al., 2008). The regions included the left DLPFC (l-DLPFC; CHs 13, 14, 16, and 17), the right DLPFC (r-DLPFC; CHs 35, 36, 39, and 40), the left ventrolateral PFC (l-VLPFC; CHs 2, 6, and 9), the right VLPFC (r-VLPFC; CHs 26, 29, and 33), the left FPA (l-FPA; CHs 3, 7, and 10), and the right FPA (r-FPA; CHs 25, 28, and 32) (Fig. 2B). LBPA40 is considered valid because optical properties of neighboring channels are known to be similar. However, with this method, optical properties in different ROIs can cause systematic bias during statistical analysis. Therefore, we limited the analyses to ROI-wise and used a false discovery rate (FDR) to control the low proportion of false positives.

### 2.6 Mood measurements

The TDMS was adopted to evaluate mood state before and after both SRUN and CON conditions. The TDMS is a momentary mood scale consisting of eight words describing arousal and pleasure states: energetic, lively, lethargic, listless, relaxed, calm, irritated, and nervous. The participants used a 6-point Likert scale ranging from 0 = not at all to 5 = extremely to describe their feelings. Subsequently, the scores were calculated and interpreted to determine arousal and pleasure levels.

### 2.7 Head acceleration measurements

An AS-20GB acceleration sensor was connected to a strain amplifier DPM-600A (both are products of Kyowa Electronic Instruments Co., Ltd.). This output signal was recorded by a personal computer after real-time conversion to a digital signal using PowerLab/16SP (ADInstruments). The sensor was attached on the top of the head with a lightweight bracket so that the acceleration detection direction was perpendicular to the floor. Very slow running was executed on a treadmill for 2 minutes. Vertical HA was measured and recorded while very slow running was executed on a treadmill for 2 minutes. The head moves up and down vertically once with each step, causing acceleration of the head. The incremental difference between the minimum and maximum acceleration values for 10 steps were standardized (see Supplemental Material 3). HA was assessed separately after the SRUN condition because the accelerometer, which was rigidly attached to the head to maintain high validity, could interfere with the pleasant sensations of running for 10 minutes.

### 2.8 Statistical analysis

The statistical analysis strategy followed the same procedure as in our previous study, which examined moderate-intensity exercise rather than very slow running (Damrongthai et al., 2021). Psychological mood state was subjected to repeated-measures two-way analysis of variance (ANOVA) with session (CON, SRUN) and time (pre, post) as within-subject factors. Behavioral Stroop performance was first tested to determine whether there was significant Stroop interference using repeated-measures three-way ANOVA with condition (neutral, incongruent), session (CON, SRUN), and time (pre, post) as within-subject factors. Then, the effect of running on Stroop task performance was analyzed using repeated-measures two-way ANOVA with session (CON, SRUN) and time (pre, post) as within-subject factors. Cortical activation during pre-sessions for CON and SRUN, which were free from any effect of running, were first examined to determine whether the Stroop interference effect could be observed using one-sample *t*-test with FDR correction. Only significant ROIs for Stroop interference were subsequently analyzed for the effect of running on prefrontal activation using repeated-measures two-way ANOVA with session (CON, SRUN) and time (pre, post) as within-subject factors followed by FDR correction. Additionally, Pearson’s correlation coefficient was adopted to analyze the relationship between HA during SRUN and changes in variables to determine whether significant changes could be observed: [(post-session) – (pre-session)] for SRUN and [(post-session) – (pre-session)] for CON. The statistical significance level was set *a priori* at *p* < 0.05. The SPSS Statistical Package version 24 (SPSS, Inc., USA) was used for statistical analyses.

The sample size was confirmed to be acceptable: post-hoc sensitivity analysis, computed using G*Power 3.1, was performed based on the current sample size (24 participants) and resulted in an alpha of 0.05 and power of 80%, demonstrating sufficient sensitivity for detecting *t*-test differences exceeding *d* = 0.60 (with a two-tailed alpha). *t*-Tests were used to analyze the differences between SRUN and CON conditions, the primary comparison for this study, and this analysis method is consistent with our previous studies (Byun et al., 2014b; Damrongthai et al., 2021; Kuwamizu et al., 2023; Yanagisawa et al., 2010).

## 3 Results

### 3.1 Verification of very slow running

During 10 minutes of very slow running, average HR and RPE were 104.84 ± 7.61 bpm and 8.50 ± 1.95 points, respectively, for males and 107 ± 9.39 bpm and 8.68 ± 1.27 points, respectively, for females. Based on the guideline of the ACSM, these values were determined to fall within the range of very light-intensity exercise (Pescatello et al., 2014). In addition, average treadmill speeds for males and females were 5.46 ± 1.77 km/h and 3.86 ± 0.87 km/h, respectively. These are also walkable speeds, but the experimenter observed that the participants were able to maintain a running form with small steps.

### 3.2 Behavioral results

**Fig. 3.**
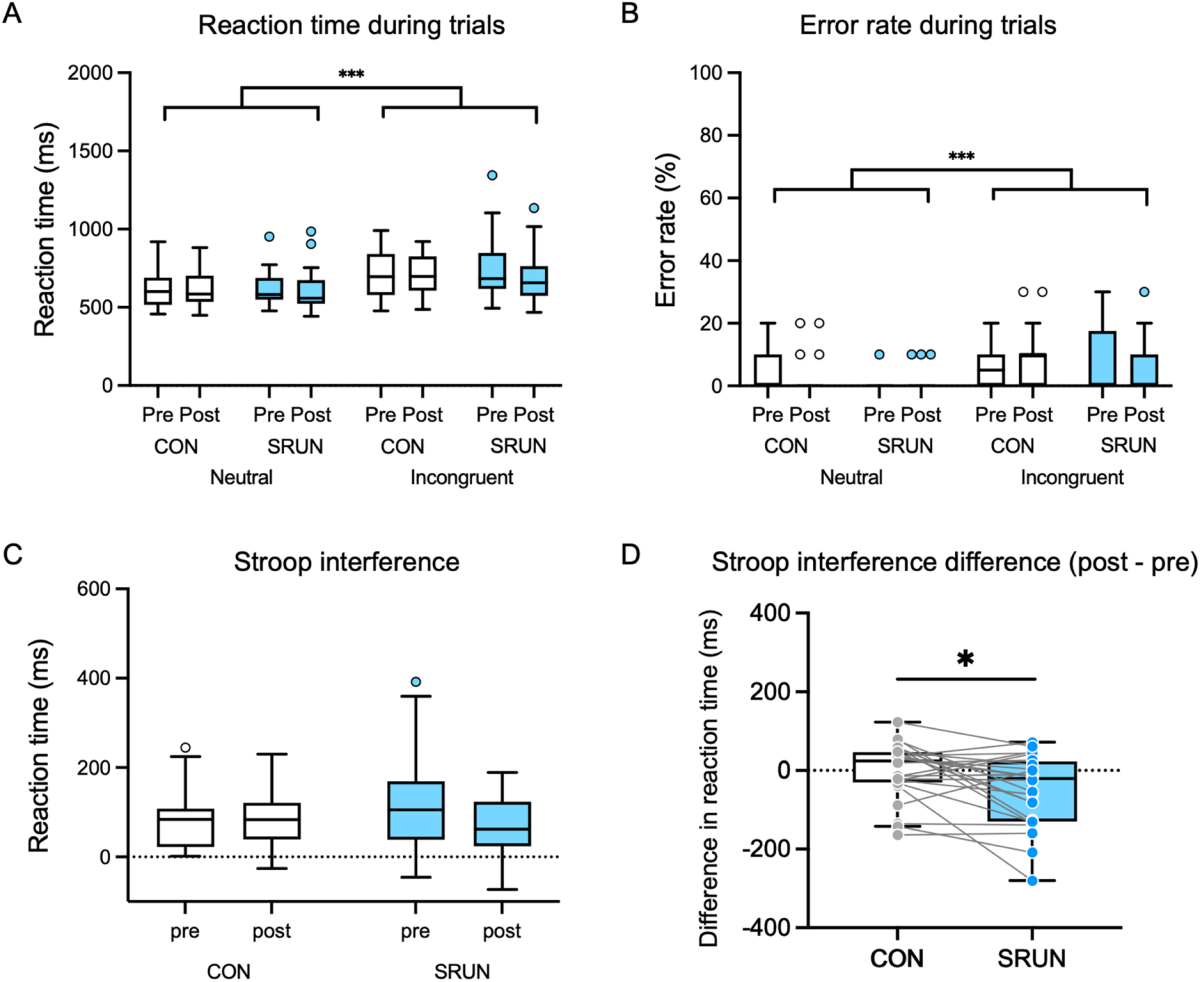
Comparison of Stroop task performance for (A) reaction time and (B) error rate between neutral and incongruent conditions. (C) Difference in Stroop interference [incongruent - neutral] between control and running sessions. (D) Contrast in Stroop interference difference ([incongruent – neutral of post-session] – [incongruent – neutral of pre-session]) between control and running. The box-and-whisker plots are drawn in the Tukey manner. Line plots represent individual data. ***p < 0.001, *p < 0.05.

RT and ER had significant main effects of condition (neutral/incongruent; *F*(1, 23) = 63.59, *p* < 0.001 (Fig. 3A) and *F*(1, 23) = 20.03, *p* < 0.001 (Fig. 3B), respectively; repeated-measures three-way ANOVA), showing that the Stroop interference effect was basically found between the neutral and incongruent conditions in all sessions of this study. Next, the effects of running on Stroop interference RT and ER were examined. Stroop interference RT revealed significant interaction between session (CON, SRUN) and time (pre, post) (*F*(1, 23) = 7.66, *p* < 0.05; repeated-measures two-way ANOVA; Fig. 3C), whereas Stroop interference ER did not reveal significant interaction (*F*(1, 23) = 2.09, *p* = 0.162). Finally, changes in Stroop interference RT were investigated and the results showed that Stroop interference RT after the SRUN session was significantly more reduced than that after the CON session (*t*(23) = 2.77, *p* < 0.05, Cohen’s *d* = 0.57; paired *t*-test; Fig. 3D).

### 3.3 Prefrontal activation

**Fig. 4.**
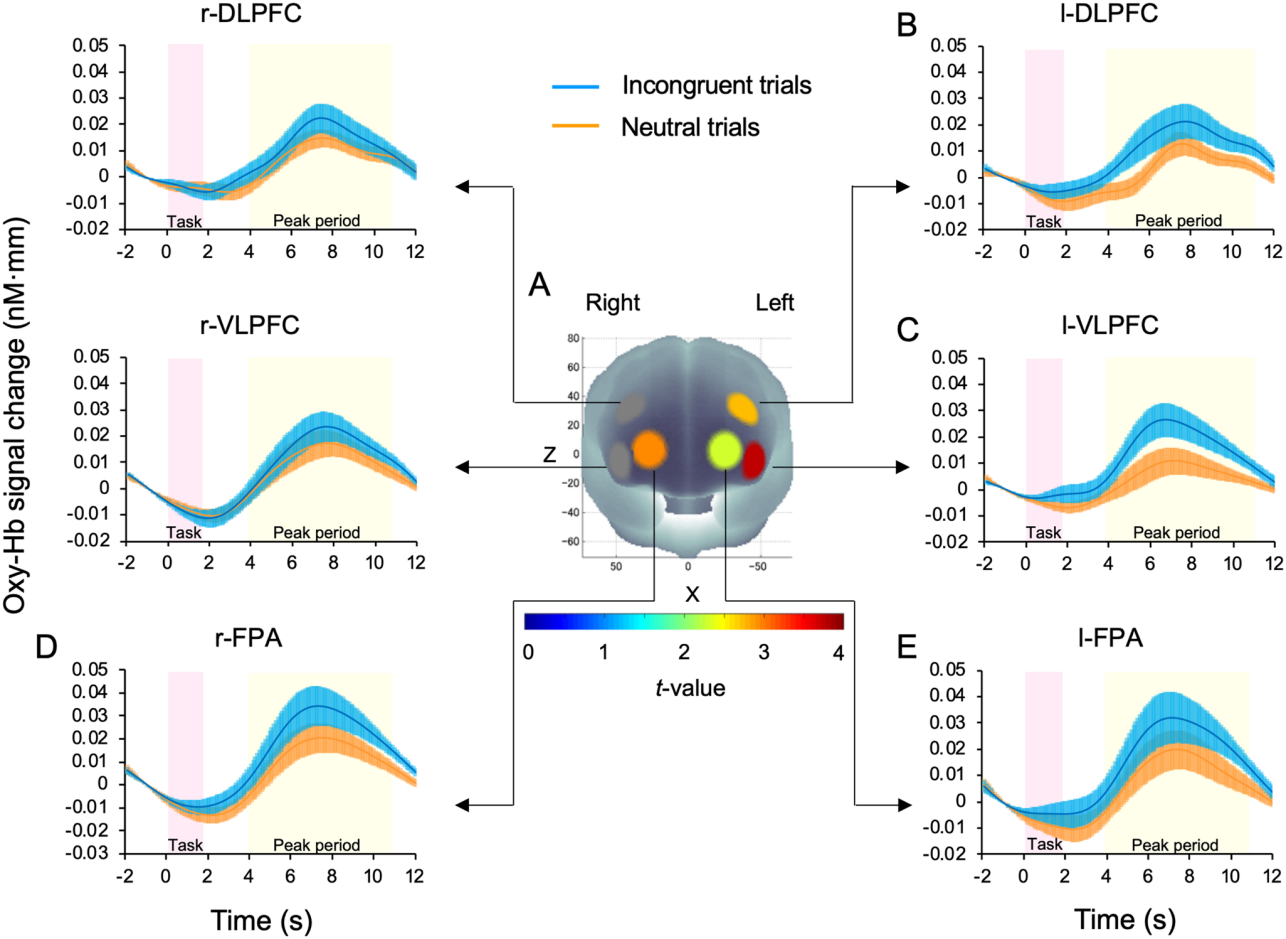
Cortical activation patterns during performance of the CWST for the CON and SRUN pre-sessions. Presented data are comparisons of averaged values between the CON pre-session and the SRUN pre-session. Baseline (2 seconds before trial onset) was set at zero and peak periods were from 4 to 11 seconds after trial onset. (A) *t*-map of (Oxy-Hb) signal change; *t*-values are as indicated by the color bar. Significant Stroop-interference-related cortical activations [incongruent - neutral] were found in 4 ROIs: the left dorsolateral prefrontal cortex (B), the left ventrolateral prefrontal cortex (C), the right frontopolar area (D), and the left frontopolar area (E). Data are mean ± SE.

**Fig. 5.**
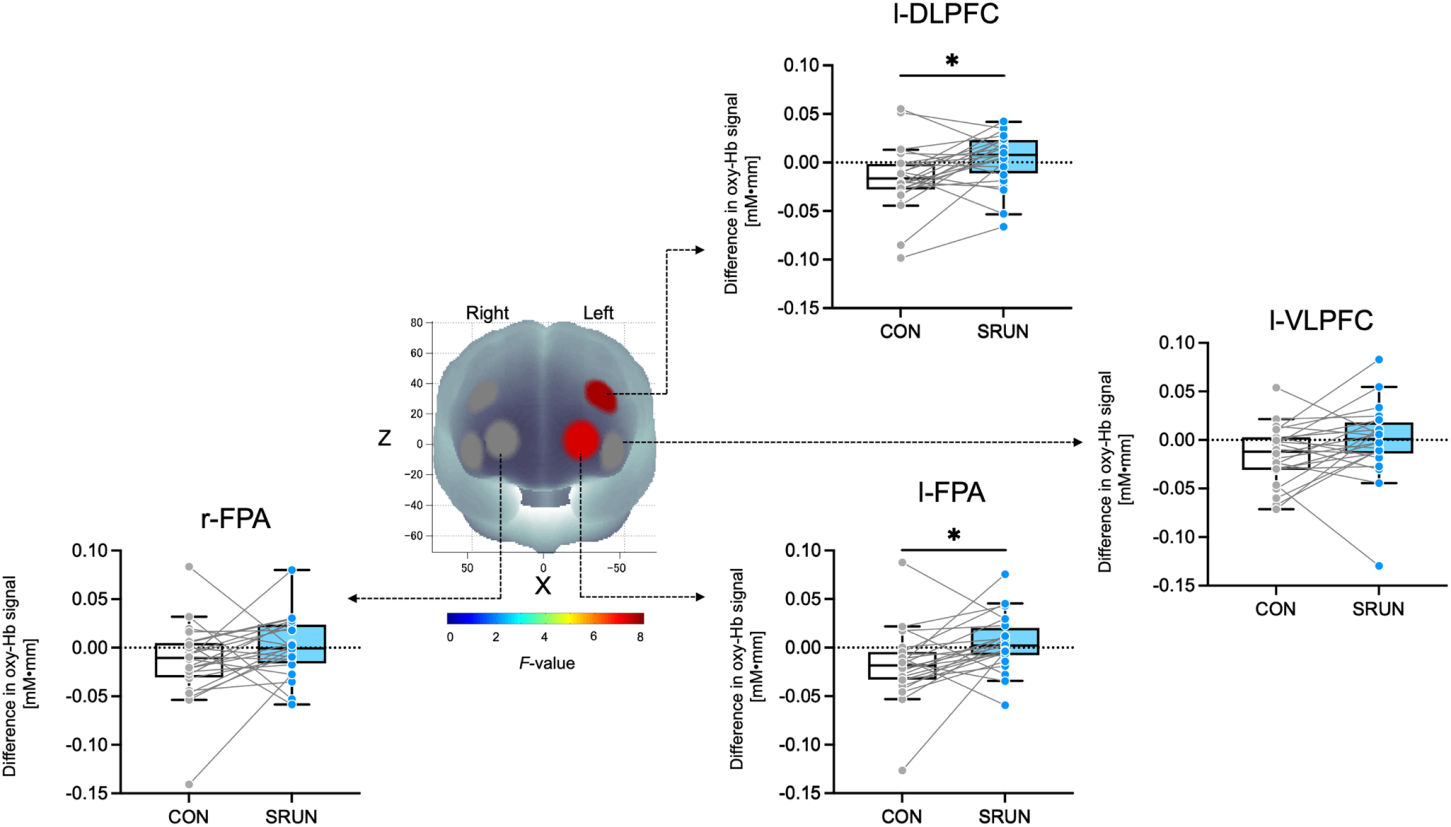
Very slow running elicits prefrontal activation in response to Stroop interference ([incongruent – neutral of post-session] – [incongruent – neutral of pre-session]). For the *F*-map of (Oxy-Hb) signal change, *F*-values are as indicated by the color bar. Among the 4 ROIs, significant differences are found in the left dorsolateral prefrontal cortex and the left frontopolar area. The box-and-whisker plots are drawn in the Tukey manner. Line plots represent individual data. *p < 0.05.

There were no significant differences in pre-session Stroop-interference-related cortical activation between CON and SRUN conditions in any ROIs. The pre-session data for the CON and SRUN sessions, which were free from any effect of exercise, were averaged and served as substrates for a ROI-wise analysis. Significant Stroop-interference-related cortical activations were found in 4 ROIs, l-DLPFC, l-VLPFC, l-FPA, and r-FPA (*p* < 0.05, one-sample *t*-test, FDR correction), indicating that the Stroop interference effect was observable in this study (Fig. 4). The effect of very slow running on prefrontal activation in these 4 ROIs was subsequently determined and the results reveal that Stroop-interference-related cortical activations had significant interactions between session (CON, SRUN) and time (pre, post) in the l-DLPFC (*F*(1, 23) = 7.73, *p* < 0.05) and the l-FPA (*F*(1, 23) = 7.34, *p* < 0.05) (repeated-measures two-way ANOVA). Finally, oxy-Hb change with Stroop interference in the l-DLPFC and the l-FPA were examined. The results reveal that the SRUN session had a significantly greater increase of oxy-Hb with Stroop interference than did the CON session in the l-DLPFC (*t*(23) = 2.78, *p* < 0.05, Cohen’s *d* = 0.57) and l-FPA (*t*(23) = 2.71, *p* < 0.05, Cohen’s *d* = 0.55) (paired *t*-test, FDR correction; Fig. 5).

### 3.4 Psychological mood

**Fig. 6.**
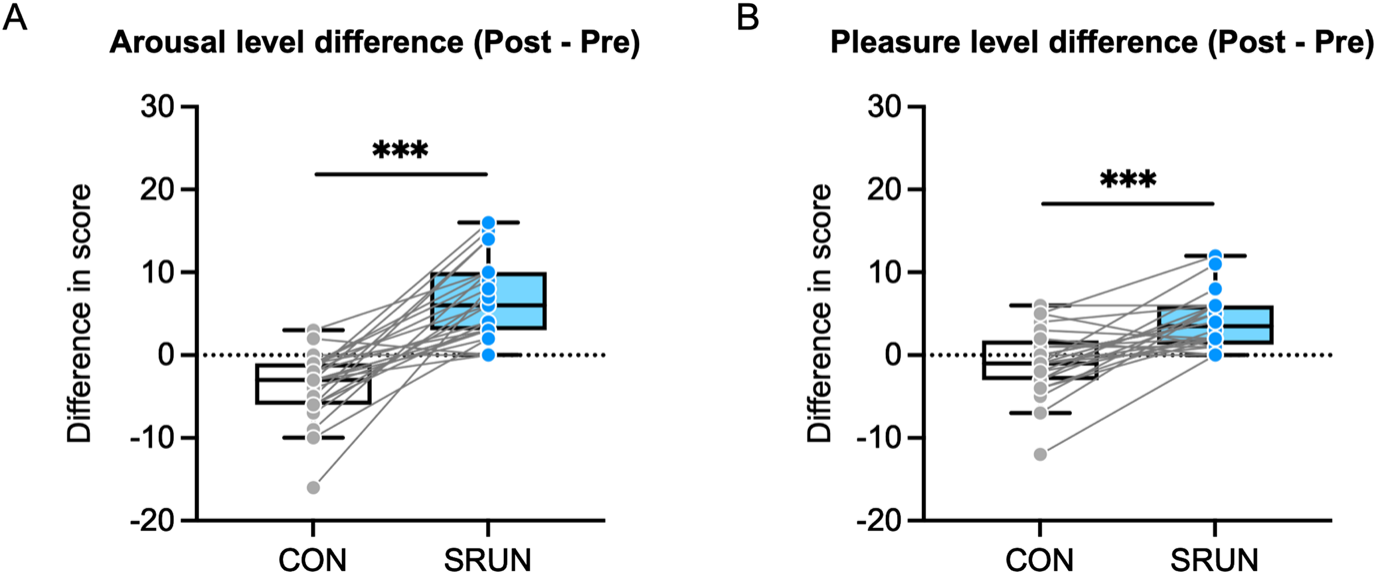
Comparison of mood change [(post-session) - (pre-session)] between CON and SRUN: (A) arousal-level differences and (B) pleasure level differences. CON = control session, SRUN = very slow running session. The box-and-whisker plot is drawn in the Tukey manner. Line plots represent individual data. ***p < 0.001.

Arousal and pleasure were found to have significant interactions between session (CON, SRUN) and time (pre, post) (*F*(1, 23) = 55.84, *p* < 0.001 and *F*(1, 23) = 22.96, *p* < 0.001, respectively; repeated-measures two-way ANOVA). Changes of arousal and pleasure were subsequently examined revealing that the SRUN session had a significantly greater increase of arousal and pleasure than did the CON session (*t*(23) = 7.47, *p* < 0.001, Cohen’s *d* = 1.53 (Fig. 6A) and *t*(23) = 4.79, *p* < 0.001, Cohen’s *d* = 0.98 (Fig. 6B), respectively; paired *t*-test).

### 3.5 Relationship between head acceleration and significantly changed variables with running

**Fig. 7.**
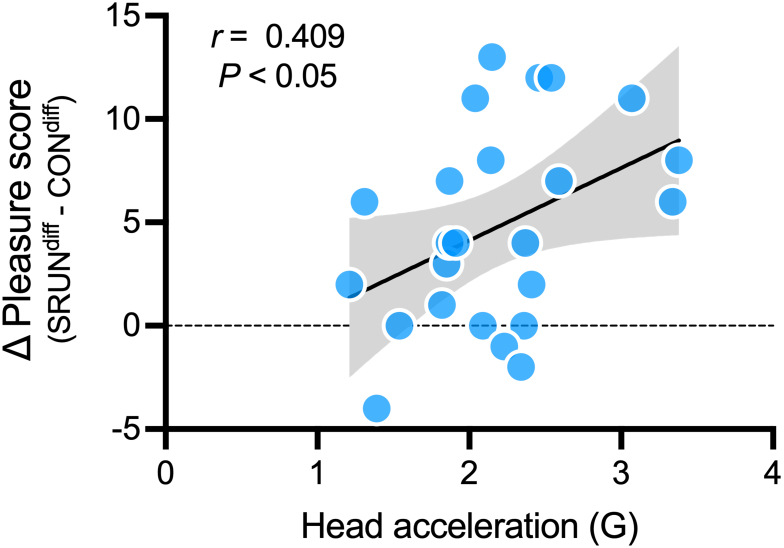
Correlation between running-generated head acceleration and running-enhanced pleasure level. The black line represents linear regression, and the gray band represents 95% confidence bands.

At the individual very slow running speed, the vertical HA averages in males and females were 2.14 ± 0.48 G and 2.22 ± 0.67 G respectively. HA influenced by very slow running was positively correlated with exercise-induced pleasure levels (*r* = 0.409, *p* < 0.05, Pearson correlation; Fig. 7). However, there was no significant association between HA and other variable changes induced by very slow running: Stroop interference RT (*r* = -0.03), l-DLPFC activation (*r* = -0.04), l-FPA activation (*r* = -0.34), and arousal level (*r* = -0.04); *p*-values for all variables were > 0.05, Pearson correlation. In addition, there was no significant correlation between HA and other potential covariate factors: treadmill speed (*r* = 0.20), *V̇*o_2peak_ (*r* = 0.17), height (*r* = -0.05), and weight (*r* = -0.22): *p*-values for all variables were > 0.3, Pearson correlation.

## 4 Discussion

The current study confirmed the hypothesis that a single bout of very slow running, a very light-intensity exercise at 35% *V̇*o_2peak_, enhances executive function with activation in lateral prefrontal subregions. Moreover, very slow running elicited an improvement in positive mood and 17% of the variance in mood enhancement was explained by inter-individual HA. The current results suggest that acute very slow running has a positive effect on both executive function enhancement and mood improvement with minimal effort.

Stroop interference (longer RT with higher ER in the incongruent condition compared to the neutral condition) was found across all sessions. Subsequently, the Stroop interference time was compared between conditions, and the results support the hypothesis that very slow running shortens Stroop interference time compared to resting control. This result is consistent with previous results on very light- or moderate-intensity pedaling (Byun et al., 2014b; Kujach et al., 2018; Yanagisawa et al., 2010) and moderate-intensity running (Damrongthai et al., 2021). Therefore, the present findings build upon the results of previous studies by suggesting that even very slow running benefits executive function.

Subsequently, we focused on investigating the neural substrates for Stroop interference. In response to Stroop interference (Incongruent – Neutral), Oxy-Hb signal significantly increased in the 4 ROIs: l-DLPFC, l-VLPFC, l-FPA, and r-FPA during pre-sessions. These results are consistent with previous neuroimaging studies, including fNIRS, electrostimulation, and lesions, which have reported that Stroop interference is consistently associated with the PFC, and more specifically l-PFC (Byun et al., 2014b; Frings et al., 2018; Kuwamizu et al., 2021; MacDonald et al., 2000; Perret, 1974; Yanagisawa et al., 2010). The effect of running on prefrontal activation was subsequently determined, and the results show that very slow running elicits significant increases in activation in the l-DLPFC and l-FPA. The l-DLPFC, a brain locus implicated in inhibitory control and mood regulation, had increased Oxy-Hb response to Stroop interference (Leung et al., 2000; MacDonald et al., 2000; Song & Hakoda, 2015). Furthermore, the FPA, a brain region which generally activates with the DLPFC in response to tasks involving manipulation and monitoring, such as planning for action, has been found to have significant activation on the left side (Christoff & Gabrieli, 2000). These results are consistent with a previous study, which demonstrated that 10 minutes of very-light-intensity pedaling elicits activation of the l-DLPFC and l-FPA with Stroop interference processing (Byun et al., 2014b; Kuwamizu et al., 2023). It is suggested that the neural basis on which very slow running improves executive function is similar to that of pedaling exercises at the same intensity. On the other hand, previous studies on acute moderate-intensity running have shown extensive activation of the r-PFC in addition to the left side with Stroop interference processing (Damrongthai et al., 2021). This difference in prefrontal activation patterns may depend on exercise intensity (very slow vs. moderate). Very slow running may have minimal hypothalamus–pituitary–adrenal axis stress responses and distinct differences in the level of activation of the arousal system compared to moderate or more intense efforts (Ohiwa et al., 2006; Soya et al., 2007), which may be related to the prefrontal activation area elicited by running. This requires further investigation.

Our previous study showed that moderate-intensity running causes mood changes related to arousal and pleasure that coincide with the facilitation of PFC function (Damrongthai et al., 2021). Based on these previous findings, we tested whether acute very slow running would improve mood. As we hypothesized, very slow running improved both arousal and pleasure levels compared to resting control as well as the moderate-intensity running of the previous study did (Damrongthai et al., 2021). Previous pedaling studies have shown that very light-intensity exercise increases psychological arousal (Byun et al., 2014b; Suwabe et al., 2018), possibly associated with the catecholaminergic system which originates from the locus coeruleus (Hiraga et al., 2024; Kuwamizu et al., 2023, 2022; Yamazaki et al., 2023). The current study also attempted to measure pupillometry and confirmed pupil dilation during running. However, the data was limited to a small sample size due to device errors related to body movement (see Supplemental Material 4). Understandably, the brain’s catecholaminergic arousal system is activated during very slow running, comparably to pedaling, and might contribute to the elicitation of PFC activation and to improved executive function.

Interestingly, previous studies have shown that very light-intensity pedaling exercise increases arousal but has only a small effect on pleasure levels (Byun et al., 2014b; Suwabe et al., 2018; Kuwamizu et al., 2023). Our exploratory analysis suggested that the positive impact of very slow running on pleasure exceeded the impact produced with a comparable intensity pedaling (Supplemental Material 5). This increase in pleasure level seen with very slow running may be characteristic of this exercise. In addition, HA, a property of running, during very slow running, was examined. Interestingly, the magnitude of HA was positively correlated only with changes in pleasure levels induced by running, and this may indicate a running-specific effect, as discussed above. Additional experiments confirmed that HA does not vary much during pedaling exercise, even if participants pedal at a high rate (Supplemental Material 6). This suggests that, in addition to the common exercise effects of pedaling and running (oxygen uptake level, HR, arousal level, and RPE are common to the two exercises), rhythmic up-and-down oscillations contribute to mood improvement. This is a new and challenging hypothesis, and the exact neural circuitry involved in the positive effect of HA on mood has not been identified and needs further investigation. A recent animal study demonstrated that HA modulates behavior and the serotonergic system in the PFC (Ryu et al., 2020). As the brain’s serotonergic system is involved in emotion regulation, this is one candidate mechanism of running- and head-acceleration-induced mood enhancement. This mechanism may work in addition to the arousal system alone (i.e., with no HA) and may form a running-specific arousal state. As other candidate factors, physiological changes due to foot strike (Lyngeraa et al., 2013; Palatini et al., 1989) and high tempo rhythm sensation with foot beats might also be related to mood changes and PFC activity (Fukuie et al., 2022, 2023). The current results represent a starting point, and further research is expected to determine how HA during running correlates with positive mood changes. It would be interesting to investigate the effects of dose-response for HA and other forms of rhythmic up-and-down oscillations such as jump rope (Yamashita & Yamamoto, 2021).

While HA may play a role in mood alterations, it does not correlate with the improvements in executive function and activation of the lateral prefrontal subregions induced by running. Interestingly, even with minimal HA during pedaling, it has been shown to positively enhance cognitive aspects, especially prefrontal executive function and hippocampal memory (Byun et al., 2014b; Suwabe et al., 2018). The lack of significant findings linking HA to enhanced prefrontal executive function implies that it might not be a critical factor in the cognitive benefits derived from exercise benefits.

To enable fNIRS measurement after running, we adopted an experimental model designed to minimize systemic influences on the cortical signal. A recurring challenge in exercise-fNIRS research is that exercise-induced non-cortical signals (skin blood flow, heart rate, and ETCO_2_ can be misread as cortical activation (Byun et al., 2014a; Miyazawa et al., 2013). Figure 1B empirically maps the decay of these peripheral signals after a 10-minute bout of very slow running, showing that all variables return to baseline within 5 minutes. This justified the 5-minute post-running delay in our protocol and offers a reproducible template for similar designs, with the required delay scaling to exercise intensity (e.g., 15 minute after moderate-intensity running at 50% *V̇*o_2peak_ (Damrongthai et al., 2021). Together, these procedures helped minimize carry-over effects of running-related non-cortical signals.

This study aimed to determine the lowest intensity/speed of running that would have a positive effect on prefrontal function and mood. The participants’ running speeds ranged from about 3 to 6 km/h. This speed is below the transition from walking to running (around 7 km/h) and is the lowest speed at which the form of running can be maintained. However, this study is the first to show that running, even at its lowest speed, is sufficient to induce a positive mood and to promote prefrontal executive function. Although this study included only healthy young adults, future validation with other populations will increase its generalizability. Even very light-intensity pedaling exercise, when maintained consistently, improves PFC function in older adults (Byun et al., 2024). It will be an interesting challenge to see if a long-term intervention of very slow running outperforms pedaling exercise.

One potential limitation is that our fNIRS apparatus lacked short-separation channels, creating a risk of contamination from superficial signals. However, we argue that this impact was minimal due to three key lines of evidence. First, our study design inherently reduced confounds. We evaluated Stroop interference (the difference between incongruent and neutral conditions), which minimizes general task-related noise (e.g., button presses). We also explicitly minimized exercise-induced physiological artifacts, such as persistent skin blood flow, through our careful protocol (Fig. 1B). Second, the data topography supports a neural origin. Systemic effects are expected to be spatially uniform (Noah et al., 2021), whereas our Stroop interference-related activation was strongly left-lateralized (l-DLPFC and VLPFC; Fig. 4). This specific localization is highly consistent with numerous fMRI and fNIRS studies (Byun et al., 2014b, 2024; Damrongthai et al., 2021; Fukuie et al., 2023; Kujach et al., 2018; Kuwamizu et al., 2021, 2023; MacDonald et al., 2000; Okayasu et al., 2023; Schroeter et al., 2002, 2004; Suwabe et al., 2021; Tak et al., 2016; Yanagisawa et al., 2010; Zysset et al., 2007), suggesting we reliably detected task-related neural signals. Finally, to further address this concern and recent methodological critiques (Yücel et al., 2025), we performed an additional sensitivity analysis. We employed the hemodynamic modality separation method to algorithmically eliminate systemic noise (Yamada et al., 2012). Our primary findings remained robust (see Supplemental Materials 7 and 8), providing support that the observed effects reflect neural activation rather than systemic artifacts. Future research utilizing advanced methods to ensure signal fidelity is encouraged to further corroborate the findings of the present study.

Second, the current discussion on the involvement of HA is limited by the study design, which included only seated rest and running conditions. Although a walking condition was beyond the scope of this study, future research should incorporate additional locomotion modes, such as walking (as introduced in Supplemental Material 6), to further examine the causal relationship between HA and its potential effects on cognition and mood.

In conclusion, the current study successfully determined the beneficial effect of a 10-minute single bout of very slow running on improving mood and executive function with lateral prefrontal neural activation. This discovery of the positive relationship between head acceleration and improved mood may be one of the underlying mechanisms through which very slow running promotes mental health. To this end, these findings contribute to social wellbeing by encouraging people with various health conditions to keep physically and mentally active, while also maintaining their own safety, through very slow running.

## Supplementary Material

Supplementary Material

## Data Availability

All data that support the findings of this study are available upon request from the corresponding author within the limits set by the Institutional Ethics Committee of the University of Tsukuba.
