# Supplementary material for: Slow running benefits: Boosts in mood and facilitation of prefrontal cortex function even at very light intensity
Source: Imaging Neurosci (Camb). 2026 Aug 4;4:IMAG.a.1297. doi: 10.1162/IMAG.a.1297 (PMC13439976; doi:10.1162/IMAG.a.1297)
Supplement: Supplementary file 1 [file IMAG.a.1297_supp.pdf]

**Supplemental Material**

# Slow running benefits: Boosts in mood and facilitation of prefrontal cortex function even at very light intensity

Chorphaka Damrongthai, Ryuta Kuwamizu, Yudai Yamazaki, Naoki Aoike, Dongmin Lee, Kyeongho Byun, Ferenc Torma, Kazuki Hyodo, Worachat Churdchomjan, Michal A. Yassa, Kazutaka Adachi, and Hideaki Soya

## Supplemental Material 1

### $\dot{V}O_{2peak}$ determination

Maximal oxygen uptake ($\dot{V}O_{2peak}$) was determined in the first phase of the experiment to determine the appropriate intensity of a very light exercise for each participant to reach 35% $\dot{V}O_{2peak}$ based on the definition of exercise intensity of the American College of Sports Medicine (American College of Sports Medicine, 2016).

A treadmill (Pulsar® h/p/cosmos, Germany) with a safety harness was used for the incremental exercise test. The test began with a 2-min rest and a 3-min warm-up at 5 km/hr (females: 4 km/hr). Then, the treadmill speed was increased by 1 km/hr every minute while maintaining the treadmill slope at 0.1% ascent until the participant reached volitional exhaustion, which was immediately followed by a 3-min cool-down to complete the test (Harling *et al.*, 2003). Heart rate (HR) and rate of perceived exertion (RPE) were recorded every minute (Borg, 1982). A gas analyzer (Aeromonitor AE300, Minato Medical Science, Japan) was used to measure ventilation parameters: oxygen consumption ($\dot{V}O_2$), carbon dioxide production ($\dot{V}CO_2$), and respiratory exchange ratio (R) at a sampling rate of 0.1 Hz (Harms *et al.*, 1998). $\dot{V}O_{2peak}$ was set once the participant met one of the following three criteria: R > 1.15, HR is $\pm$ 10 bpm of age-predicted maximal HR, or RPE = 19-20 (Hanson *et al.*, 2016). Physiological parameters and treadmill speed at volitional exhaustion are shown in Table S1.

To define the exertion needed to achieve 35% $\dot{V}O_{2peak}$, $\dot{V}O_2$ (x-coordinate) was plotted against treadmill speed (y-coordinate). Then, the treadmill speed at 35% $\dot{V}O_{2peak}$ was determined by a simple linear regression formula that was calculated using Microsoft

Excel (Morgan DW Baldini FD Martin PE, 1989). The resulting treadmill speed for each participant was assigned as their exertion level during the 10 min of running. As the 35% $\dot{V}O_{2peak}$ running speed was a minimal running speed at which it was possible to walk, determination of intensity using a simple linear regression based on the treadmill maximal graded exercise test may have validity limitations. To maintain the validity of the participant-tailored 35% $\dot{V}O_{2peak}$ running speeds, HR was monitored for the first 3 minutes of the 10-min running exercise and the treadmill speed was adjusted if the participant was prominently below or above their target HR for 35% $\dot{V}O_{2peak}$. HR at 35% $\dot{V}O_{2peak}$ was determined by plotting $\dot{V}O_2$ (x-coordinate) against HR (y-coordinate).

**Table S1.** Physiological parameters and final treadmill speed at volitional exhaustion (n=24).

| | **Male (n=13)** | **Female (n=11)** |
|---|---|---|
| Maximal HR (bpm) | 191.15 ± 8.43 | 190.00 ± 7.25 |
| RPE | 19.23 ± 0.60 | 19.27 ± 1.01 |
| R | 1.14 ± 0.03 | 1.13 ± 0.06 |
| $\dot{V}O_{2peak}$ (ml/kg/min) | 48.24 ± 6.25 | 40.03 ± 3.84 |
| Final treadmill speed (km/h) | 16.54 ± 2.40 | 13.55 ± 1.97 |

Values are mean ± SD, HR = heart rate, RPE = rate of perceived exertion, R = respiratory exchange ratio, $\dot{V}O_{2peak}$ = maximal oxygen uptake

**Supplemental Material 2**

**Investigation of non-cortical physiological changes induced by very slow running**

Exercise basically induces whole-body physiological changes. To measure cortical hemodynamics after very slow running using Functional near-infrared spectroscopy (fNIRS), it was necessary to determine how non-cortical physiological parameters, such as skin blood flow (SBF) and HR, change in order to specify appropriate fNIRS measurement timing after very slow running and to eliminate possible signal contamination (Byun *et al.*, 2014*a*).

A laser-Doppler probe (FLO-C1, OMEGAWAVE, Japan) was attached at the Fpz of the international 10-20 system to assess SBF. End-tidal carbon dioxide ($ETCO_2$), which reflect the level of carbon dioxide released at the end of an exhalation, was measured using a gas analyzer (Aeromonitor AE300, Minato Medical Science, Japan) (Richardson *et al.*, 2016). The experiment protocol started with 3 min of rest, continuing with 10 min very light-intensity running and then rest again for 20 min. SBF, HR, $\dot{V}O_2$ and $ETCO_2$ were monitored every minute throughout the experiment. Seven Japanese young adults participated in this aspect of the study as shown in Table S2. No participant reported smoking, a history of neurological disorders, or a condition requiring medical care.

The participants were able to perform very light-intensity exercise or very slow running based on the ACSM 2017 criteria. The physiological responses and treadmill speed during the 10 min of very slow running are shown in Table S3. Non-cortical physiological changes induced by very slow running are shown in Figure S1.

Examining the non-cortical physiological changes that were measured throughout the experiment, it was shown that SBF had a non-significant increase and that HR and $ETCO_2$ both ha d similar responses in that significant increase was found from the beginning of very slow running until 2 min into the recovery period. It was clear that all non-cortical physiological variables changed during very slow running and completely returned to baseline within 5 min into the recovery period. Thus, we determined that 5 min after very slow running was an appropriate time to start measuring cortical hemodynamics with fNIRS while avoiding possible signal contamination.

**Table S2.** Participant characteristics (n = 7)

| | **Male (n = 4)** | **Female (n = 3)** |
|---|---|---|
| Age (yr) | 24.25 $\pm$ 2.62 | 20.66 $\pm$ 0.57 |
| Weight (kg) | 62.10 $\pm$ 8.18 | 45.06 $\pm$ 1.86 |
| Height (cm) | 166.5 $\pm$ 5.06 | 159.67 $\pm$ 4.93 |
| BMI (kg/m$^2$) | 22.45 + 3.21 | 17.70 + 1.45 |
| $\dot{V}O_{2peak}$ (ml/kg/min) | 52.04 + 7.56 | 39.59 $\pm$ 2.83 |

Values are mean $\pm$ SD; $\dot{V}O_{2peak}$ = maximal oxygen uptake

**Table S3.** Physiological responses and treadmill speeds during 10 min very slow running (n = 7)

| | **Male (n = 4)** | **Female (n = 3)** |
|---|---|---|
| $\dot{V}O_2$ (% $\dot{V}O_{2peak}$) | 36.36 $\pm$ 3.47 | 40.64 $\pm$ 5.71 |
| HR (%$HR_{max}$) | 55.11 $\pm$ 2.36 | 58.70 $\pm$ 4.30 |
| RPE | 8.00 $\pm$ 1.78 | 9.77 $\pm$ 2.41 |
| Treadmill speed (km/h) | 5.00 $\pm$ 1.47 | 3.16 $\pm$ 0.28 |

Values are mean $\pm$ SD, $\dot{V}O_2$= oxygen uptake, $\dot{V}O_{2peak}$ = maximal oxygen uptake, HR = heart rate, $HR_{max}$ = maximal heart rate, RPE = rate of perceived exertion

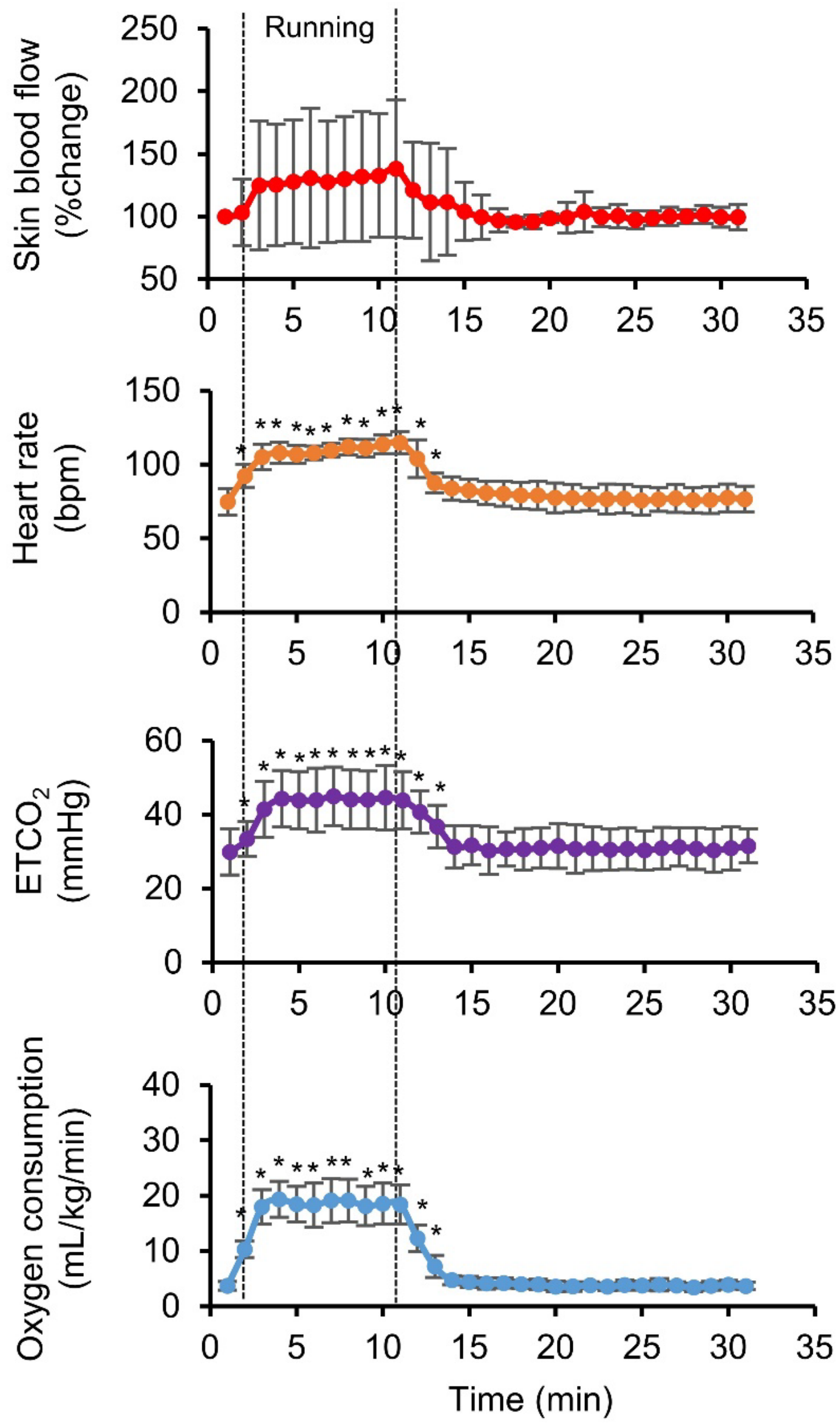


**Figure S1.** Illustrations of the non-cortical physiological parameters at baseline (set at 100%), during 10 min of very slow running and 20 min rest of. Inter-subject means at each time point were plotted; error bars indicate standard deviation. Time points with significant change compared to baseline are shown with asterisks ($p < 0.05$, one-way ANOVA with Dunnett correction).

## Supplemental Material 3

### Head acceleration measurement during very slow running

One rodent study has reported that Head acceleration (HA), the rate of change in head velocity, could induce the internalization of serotonin receptors in the prefrontal cortex (PFC) (Ryu *et al.*, 2020). Given running as a locomotive exercise that could generate HA, in particular vertical HA (the change of head velocity in up and down directions), it was very challenging to set up an experiment to investigate this parameter during human running. An accelerometer (AS-20GA; Kyowa Electronic Instruments, Japan) and its signal conditioner (CDV-700A; Kyowa Electronic Instruments, Japan) were used to investigate vertical HA during very slow running for 2 minutes. Before the experiment, the top of each participant's head was equipped with the sensors using a special attachment as shown in Figure S2.

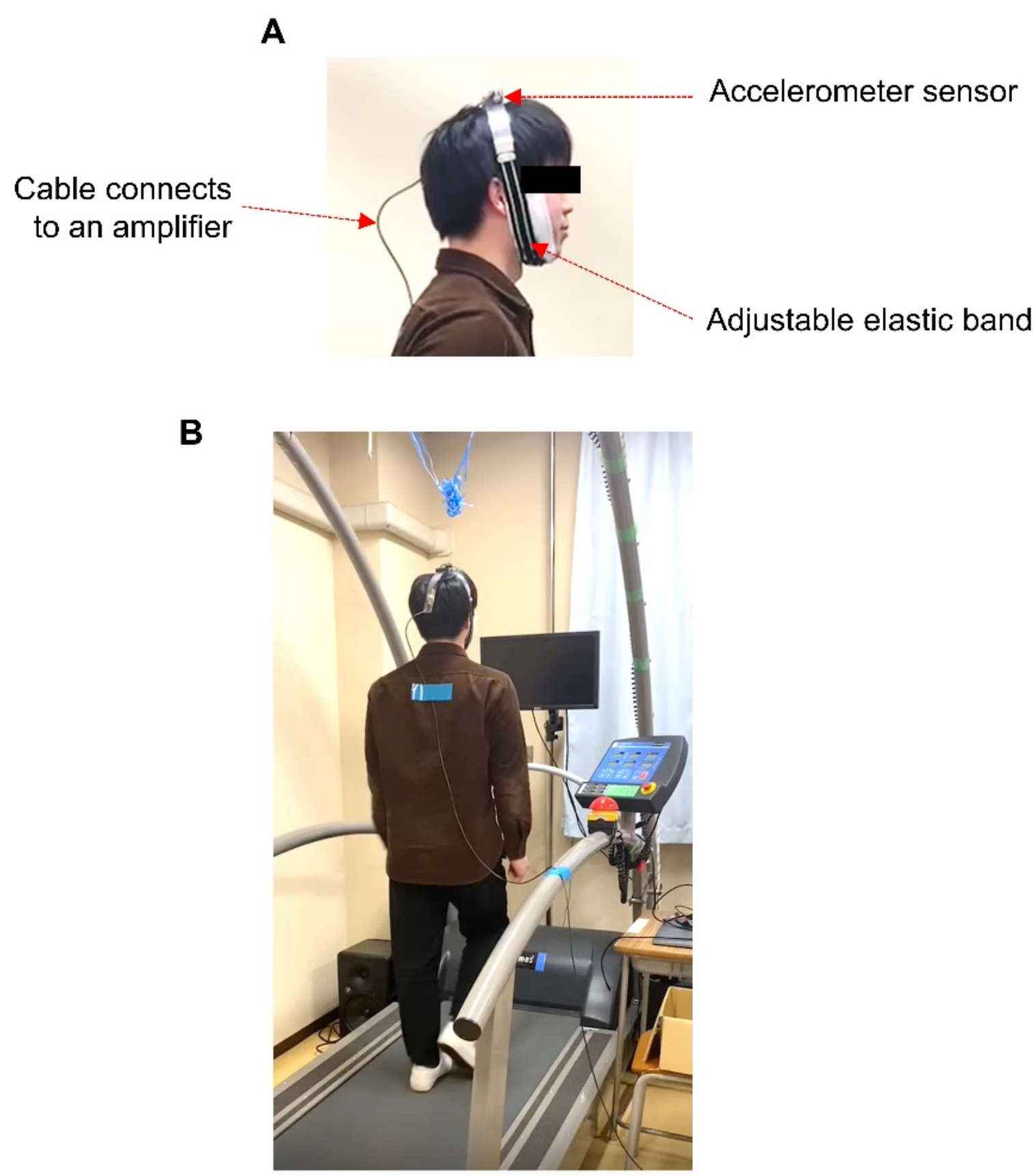

**Figure S2.** (A) Photograph of the accelerometer sensor that was attached to the top of each participant's head. (B) Photograph of the experimental setup for HA measurement during very slow running.

The output voltage (1G = 0.2 V) from the measuring system was processed with an A/D converter (PowerLab/16SP of ADInstrument, New Zealand) and recorded using a personal computer. Then, vertical acceleration, which was reported in units of gravitational acceleration (G), was subjected to analysis as shown in Figure S3. The results of the ten waveforms showed that very slow running generated HA of 2.14 $\pm$ 0.67 G and 2.22 $\pm$ 0.67 G in males and females, respectively.

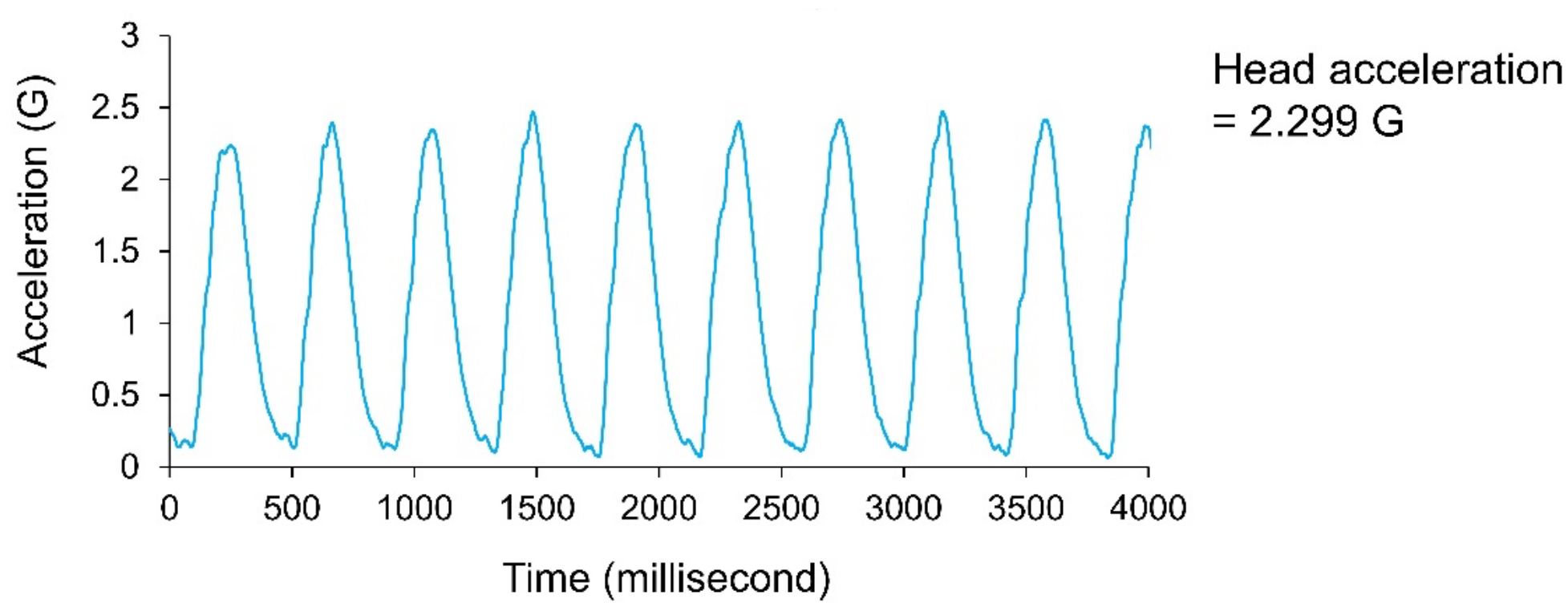


**Figure S3.** Vertical HA of one participant during very slow running.

## Supplemental Material 4

### Investigation of pupil diameter during SRUN and CON sessions

Pupil size has been considered a reliable indicator of the ascending arousal system, including locus coeruleus-noradrenaline and acetylcholine activity (Joshi *et al.*, 2016; Reimer *et al.*, 2016), and cortical state (McGinley et al., 2015; Schwalm and Jubal, 2017). We have already demonstrated that very light-intensity pedaling dilates the pupil (Kuwamizu *et al.*, 2022) at a magnitude that predicts exercise-related arousal and prefrontal executive function improvement (Kuwamizu *et al.*, 2023). In addition, using melamine imaging, we have suggested that very light-intensity pedaling-induced pupil dilation likely depends on the locus coeruleus (LC) (Yamazaki *et al.*, 2023). These findings indicate that LC-noradrenergic system regulation governs the cognition-enhancing effects of very light-intensity pedaling. However, to the best of our knowledge, no study to date has revealed pupil diameter changes during running in humans. Thus, we attempted to measure pupil dilation during running in this experiment.

The ViewTracker3 (DITECT Corporation, Japan) was used to measure pupil diameter during 10 min of very slow running exercise and 10 min of resting control. In the experimental room, light intensity was maintained at 960 Lux by covering all windows with black paper. Before the experiment, the participants were asked to wear the pupil core headset. The positions of the right and left eye cameras of the headset were adjusted to ensure that the device could measure pupil diameter properly as shown in Figure S4A. After 30 sec of calibration, the ViewTracker3 was ready to use. The experiment began with 2 min of rest followed by 10 continuous min of very slow running or of resting. The participants were asked to focus on a monitor in front of them as much as they could. The Two-Dimensional Mood Scale (TDMS) questionnaire and an input for their rate of perceived exertion (RPE) were displayed on the monitor at the 5$^{th}$ and 10$^{th}$ min to evaluate their mood state and the subjective exercise intensity. Aside from when the TDMS and RPE were displayed, the monitor displayed a gray screen throughout the experiment, as shown in Figures S4B and S4C.

For the analysis of pupil diameter, the ViewTracker3 was set to an automatic sampling frequency of 120 Hz, and reported pupil diameter in millimeters (mm). The analysis started with preprocessing of the individual data. A band-pass filter was used to remove noise. Then, the data was compressed and then smoothed by exponential moving

average (EMA). Average pupil diameter during the 2 min baseline resting and during 10 min of very slow running or of resting control were sampled and subsequently analyzed.

In several participants, the device detected an error and automatically stopped measuring during running, which might have been caused by large motions. Due to this technical issue, only the complete datasets for 11 participants were ultimately chosen to for analysis. However, the results were in accordance with our hypothesis in that significant interaction between session (CON, SRUN) and time (pre, post) was found ($F(1, 10) = 6.26$, $p < 0.05$, repeated-measures two-way ANOVA) as shown in Figure S5A. Changes of pupil diameter were subsequently examined revealing that the SRUN session had a significantly greater increase of pupil diameter than did the CON session ($t(10) = 2.48$, $p < 0.05$, Cohen's $d = 0.75$ as shown in Figure S5B. The pupil dilation during running seems to be comparable to the pupil dilation observed during our previous pedaling exercise studies (Kuwamizu et al., 2023, 2022; Yamazaki et al., 2023). It is necessary to reconfirm these results with a larger sample size and examine the relationships of pupil dilation with other variables and differences in other exercise modes in the future.

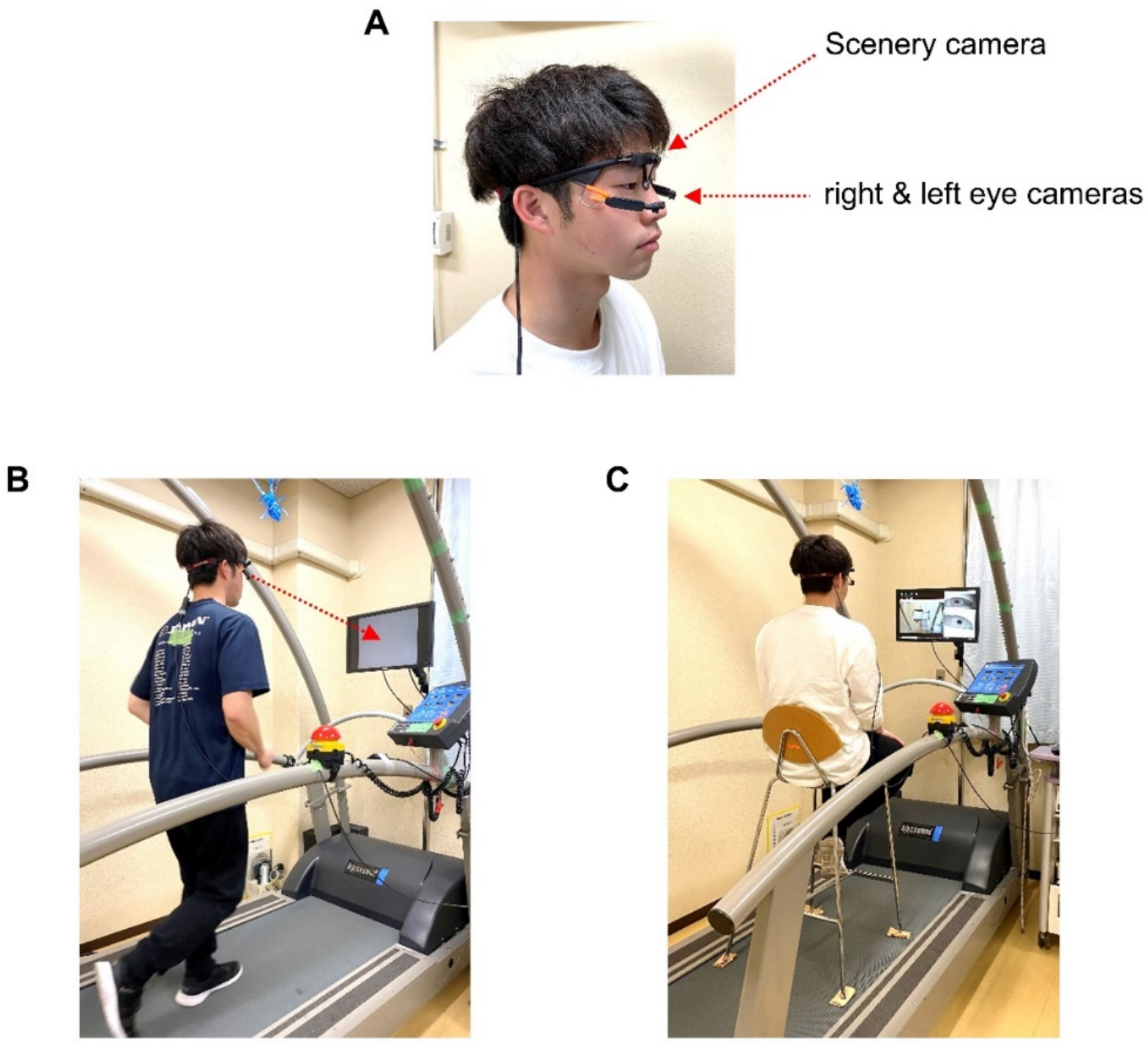


**Figure S4.** (A) Illustration of pupil core headset. A scenery and eye cameras set in the proper positions. (B) Pupil diameter measurement during SRUN session. The monitor was gray, and the participants were asked to keep focusing on it throughout the experiment. (C) Pupil diameter measurement during CON session.

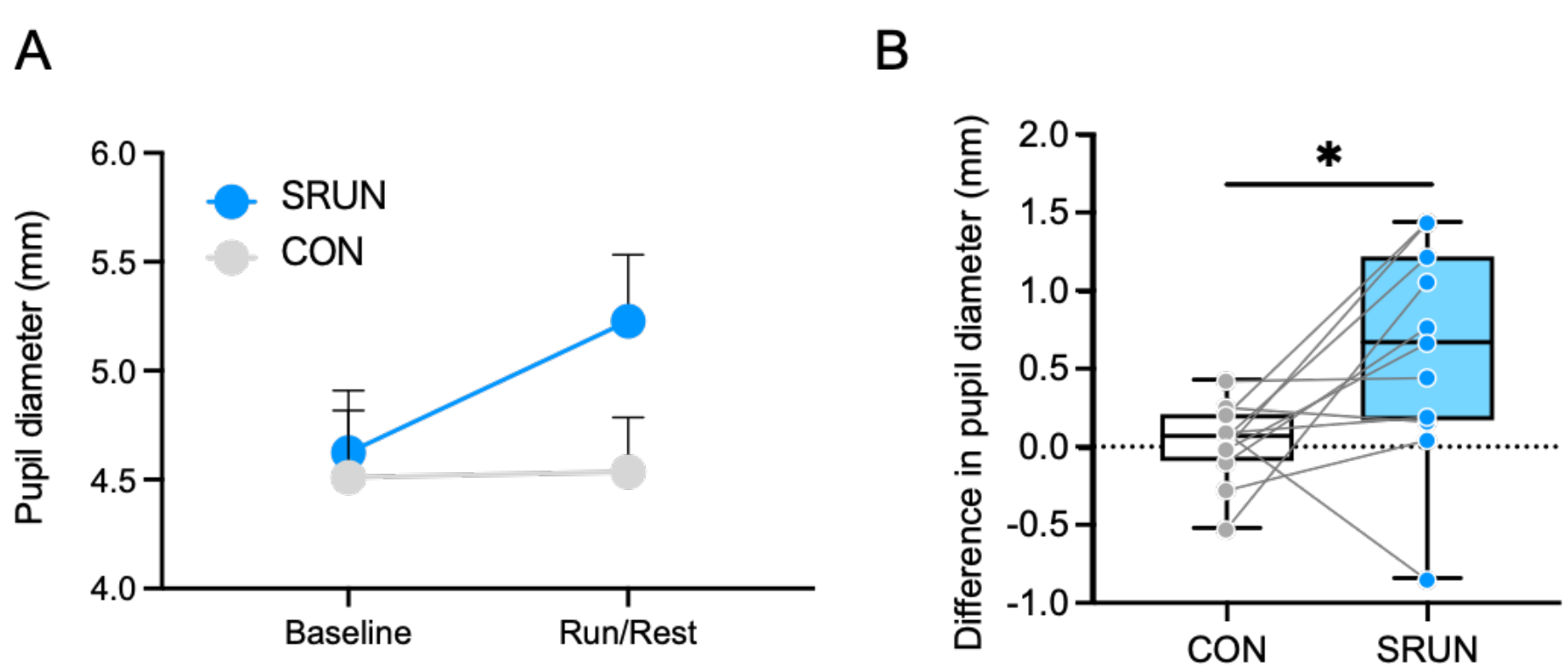

**Figure S5.** (A) Changes in pupil diameter for both CON and SRUN sessions. Data are mean ± SE. (B) Comparison in pupil diameter changes [(during run/rest) - (baseline)] between CON and SRUN sessions. The box-and-whisker plot is drawn in the Tukey manner. Line plots represent individual data. * = $p < 0.05$

## Supplemental Material 5

### Exploratory analysis for comparison with mood changes in pedaling studies

Since this study did not use pedaling exercise, the running effect was not directly compared with pedaling. However, here we conducted an unpaired t-test to explore the differences between the current running results and all individual data (n = 90) of our group's four previous pedaling studies with a similar protocol (i.e., 10 min very-light intensity exercise vs. resting control and mood states measured using the Two-Dimensional Mood Scale; TDMS) (Byun et al., 2014b; Kuwamizu et al., 2023; Suwabe et al., 2018; Yamazaki et al., 2023) as an exploratory analysis. In two of the pedaling studies (Kuwamizu et al., 2023; Yamazaki et al., 2023), mood measurements were taken immediately before the end of exercise and after the exercise, so the time at which the mean difference (Exercise - Control) reached its peak was used for analysis. The results show that the running effect (exercise (post-pre) – resting control (post-pre)) on pleasure levels seemed to be larger than the effect observed from the previous data mix from the four pedaling studies ($t (112) = 3.31$, $p < 0.01$ vs. all samples from previous four studies) as shown in Figure S6A, but not on arousal levels (($t (112) = 1.67$, $p = 0.1$), as shown in Figure S6B. This exploratory analysis supports that very slow running should benefit mood more than very light-intensity pedaling.

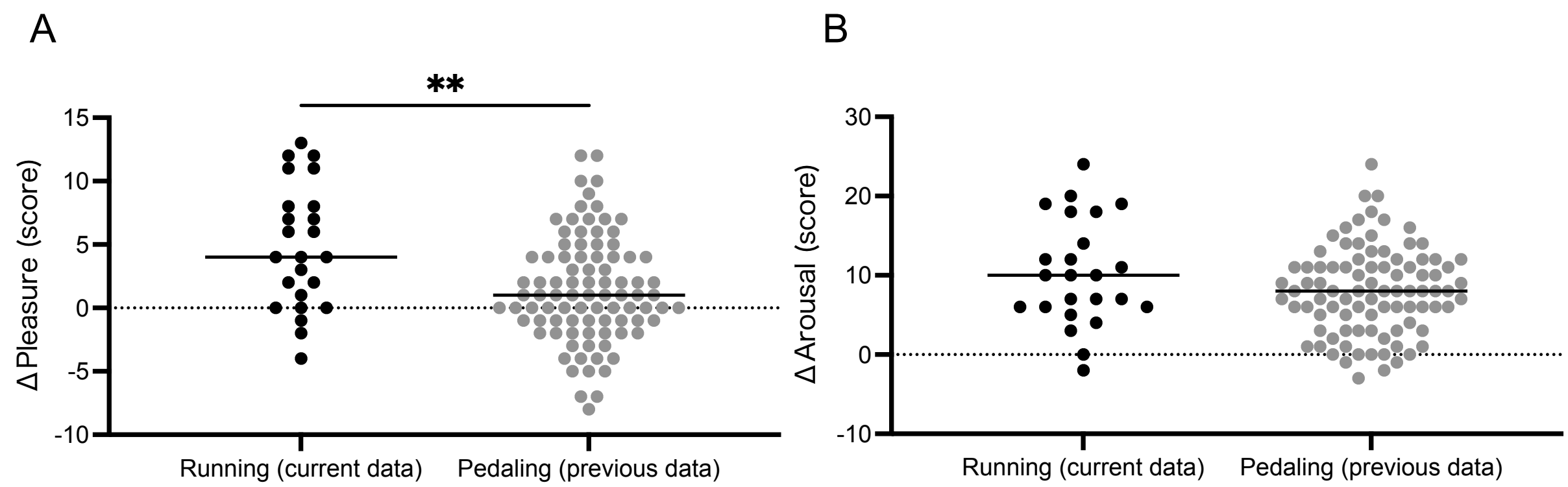


**Figure S6.** (A) Changes in pleasure [exercise (post-session) - resting control (pre-session)] for both running (current data) and pedaling (previous data). (B) Changes in arousal for both running (current data) and pedaling (previous data). Lines indicate medians. ** = $p < 0.01$

## Supplemental Material 6

### Head acceleration during pedaling, walking, and running

To investigate whether head acceleration (HA) is a feature of locomotion exercise, particularly running, we quantified HA for running, walking, and recumbent pedaling, respectively.

Five Japanese males participated in the preliminary study: mean age 25.20 ± 2.49 years [range 23-29], weight 64.90 ± 8.04 kg, height 171.58 ± 1.53 cm, and $\dot{V}O_{2peak}$ 50.43 ± 4.17 ml/kg/min. First, the HA measurement devices were put on their heads as shown in Figure S3. Then, the experiment was started with pedaling exercise at different intensities: very light intensity (35% $\dot{V}O_{2peak}$ at 60 and 100 rpm) and moderate intensity (50% $\dot{V}O_{2peak}$ at 60 and 100 rpm). After that, they were asked to walk at different speeds: 3 km/h, 4.8 km/h, and at individual very slow running speeds. Finally, they were asked to run at a very light-intensity or very slow running speed (35% $\dot{V}O_{2peak}$) and a moderate-intensity speed (50% $\dot{V}O_{2peak}$) as shown in Figure S7. Each intensity of exercise for pedaling used a cycle ergometer (Corival Recumbent, Lode, The Netherlands), walking and running were executed for 2 minutes each with 5-min rest periods between intensities.

The calculated loads for pedaling and speeds for walking and running are shown in Table S4.

**Table S4.** Exercise loads for pedaling and speeds for walking and running for each participant

| ID | Pedaling (watts) | | Walking (km/h) | | | Running (km/h) | |
|---|---|---|---|---|---|---|---|
| | 35% $\dot{V}O_{2peak}$ (very light intensity) | 50% $\dot{V}O_{2peak}$ (moderate intensity) | Speed 1 | Speed 2 | Very slow running speed | Very slow running speed (35% $\dot{V}O_{2peak}$) | 50% $\dot{V}O_{2peak}$ (moderate intensity) |
| 01 | 87 | 125 | 3 | 4.8 | 5.5 | 5.5 | 7.5 |
| 02 | 80 | 112 | 3 | 4.8 | 6 | 6 | 9 |
| 03 | 95 | 133 | 3 | 4.8 | 4.5 | 4.5 | 7 |
| 04 | 47 | 78 | 3 | 4.8 | 3.5 | 3.5 | 6.5 |
| 05 | 95 | 137 | 3 | 4.8 | 5 | 5 | 8 |

km/h = kilometers per hour; $\dot{V}O_{2peak}$ = maximal oxygen uptake

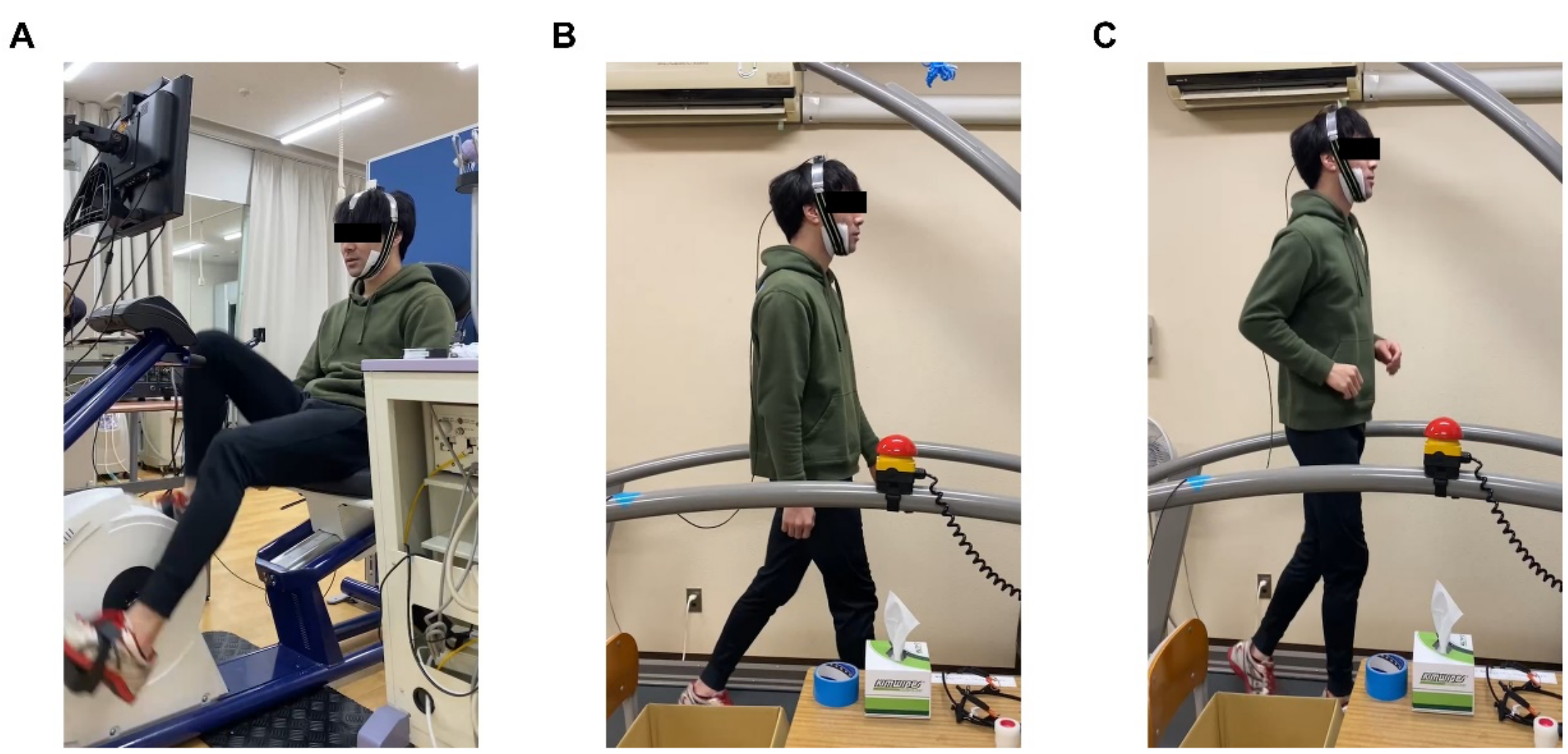


**Figure S7.** Experiment scenes for investigating vertical head acceleration; (A) pedaling exercise, (B) walking, and (C) running.

The results show that the amplitude of vertical HA during pedaling was almost undetectable as long as the pedaling was done at 60 rpm. However, even at very high pedaling rates (100 rpm) HA was slight, as shown in Figure S8. Furthermore, HA during walking was also found to be less than half that of running. Thus, the results show that vertical HA during running is greater than that during pedaling and walking.

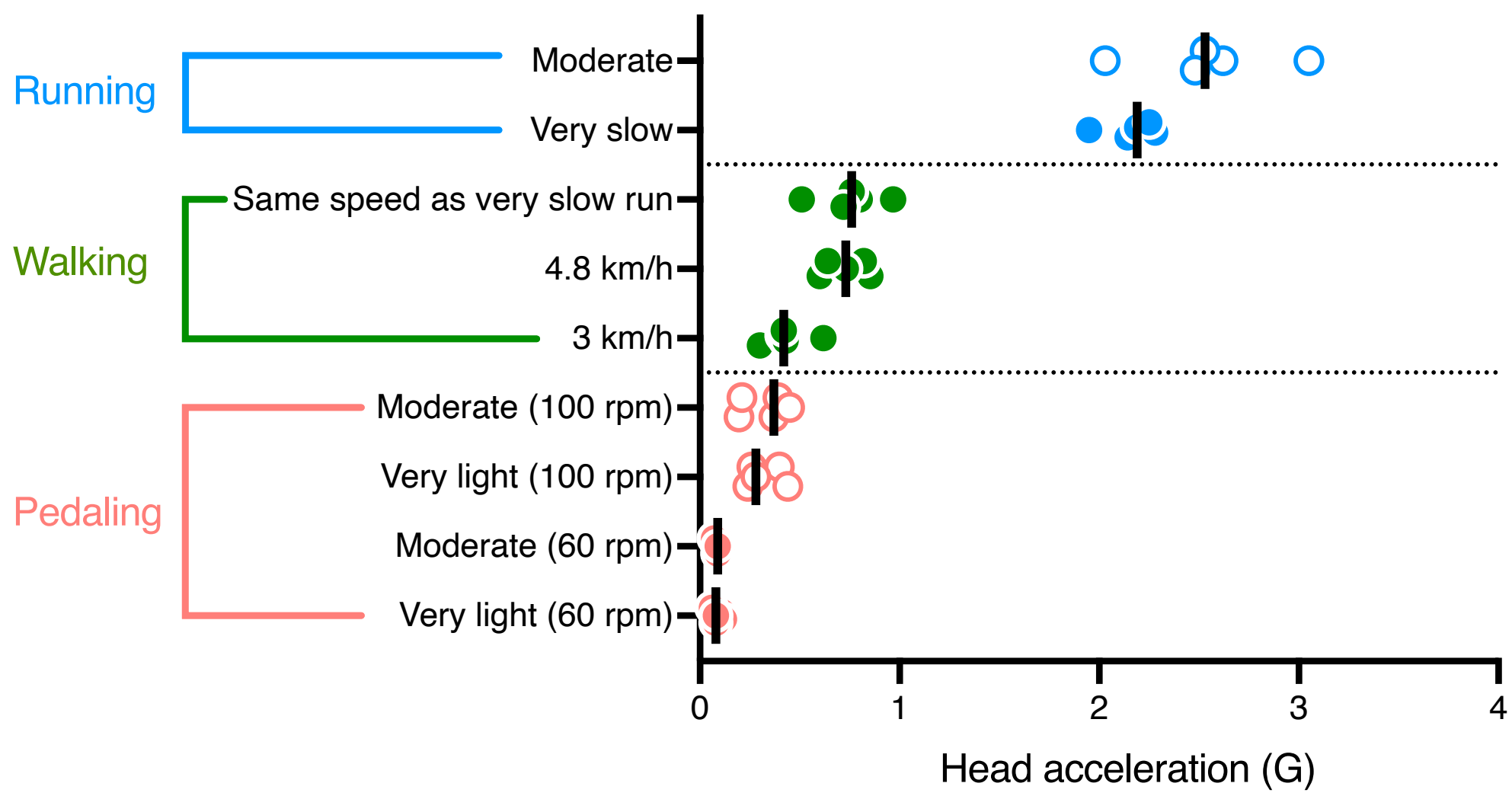


**Figure S8.** The vertical head acceleration during different modes of exercises: running, walking, and pedaling, with different intensities. Lines indicate median values; plots indicate values for each individual.

## Supplemental Material 7

### Sensitivity analysis using hemodynamic modality separation

To address recent concerns regarding methodological variability in fNIRS analysis pipelines and its potential impact on study reproducibility (Yücel *et al.*, 2025), we performed a sensitivity analysis to validate the robustness of our findings against potential systemic confounds. We employed the hemodynamic modality separation method (Yamada *et al.*, 2012), which is designed to isolate the functional (neurally-driven) signal from systemic physiological noise (e.g., superficial blood flow), even without short-separation channels. This method operates on the physiological principle that functional activation is characterized by an anti-correlation between Oxy-Hb and Deoxy-Hb, whereas systemic noise exhibits a positive correlation. We re-calculated the Post-Pre change in Stroop interference-related activation using this separation method (sensitivity analysis) and compared these results to those from our primary analysis (main analysis).

To test whether the exercise effect was dependent on the analysis pipeline, a 2 (analysis: main analysis vs. sensitivity analysis) x 2 (condition: CON vs. SRUN) repeated-measures ANOVA was conducted (using lmer in R) on the Post-Pre change values for our ROIs. The analysis revealed the significant main effect of condition (CON vs. SRUN) remained robust for both the l-DLPFC ($F(1, 69) = 16.77$, $p < 0.001$) and the l-FPA ($F(1, 69) = 12.87$, $p < 0.001$). There were no significant main effect of analysis method (main analysis vs. sensitivity analysis) for either the l-DLPFC ($F(1, 69) = 0.005$, $p = 0.94$) or the l-FPA ($F(1, 69) = 0.11$, $p = 0.75$), and also no significant analysis method x condition interaction for either the l-DLPFC ($F(1, 69) = 0.75$, $p = 0.39$) or the l-FPA ($F(1, 69) = 0.43$, $p = 0.52$). No significant interactions were observed in any other ROIs. This lack of interaction confirms that the pattern of the exercise effect (CON vs. SRUN) was statistically consistent across both analysis methods. Additionally, simple main effects analysis, estimated from the overall model using the emmeans package, confirmed this pattern. The exercise-induced increase in activation (CON < SRUN) was significant when analyzed with the main analysis (l-DLPFC: $F(1, 69) = 12.31$, $p < 0.001$; l-FPA: $F(1, 69) = 8.99$, $p < 0.01$) and remained significant when analyzed with the sensitivity analysis (l-DLPFC: $F(1, 69) = 5.21$, $p < 0.05$; l-FPA: $F(1, 69) = 4.31$, $p < 0.05$).

This sensitivity analysis confirms the robustness of our primary findings. Although the hemodynamic separation method has its own assumptions and limitations, its

application here, demonstrating no statistical interaction and preserving the main effect of running, strongly supports our conclusion that the observed exercise impact reflects genuine neural activation rather than an artifact of systemic confounds.

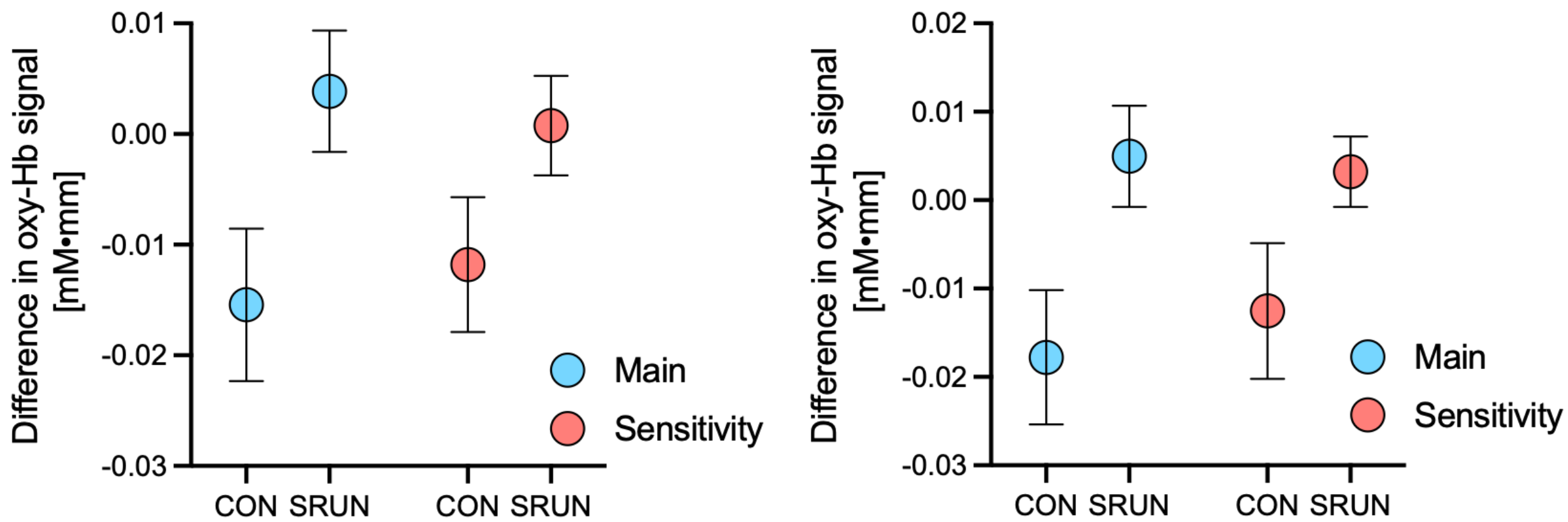


**Figure S9.** Comparison of analysis methods for calculating activation changes. The plots compare the activation changes calculated by the current data analysis (shown in light blue) and the hemodynamic modality separation method (shown in red). All data points represent the mean ± SE. In both brain regions, the activation pattern–showing higher activation after SRUN–was consistently observed for both analysis methods.

## Supplemental Material 8

## Deoxy-Hb time course patterns

We present the group-level time courses of both oxy-Hb and deoxy-Hb in the six ROIs corresponding to Figure 4 during performance of the CWST for the CON and SRUN pre-sessions.

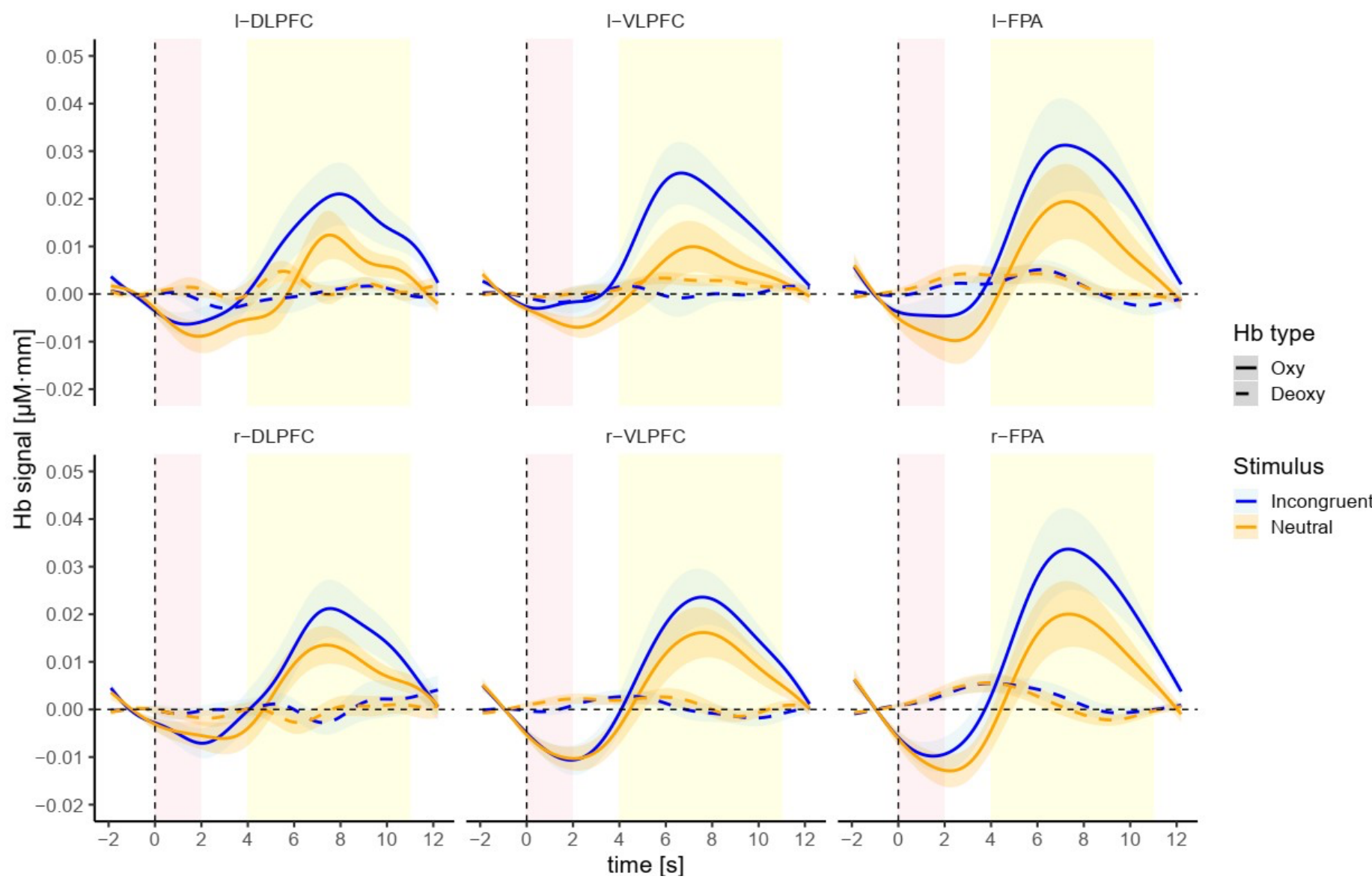


**Figure S10.** Group-level time courses of oxy-Hb (solid) and deoxy-Hb (dashed) for the Incongruent (blue) and Neutral (orange) conditions in the six ROIs. Shaded bands indicate ± SE; vertical shaded windows indicate the task (pink) and peak (yellow) periods

## References

American College of Sports Medicine, 2016. ACSM's guildlines for exercise testing and prescription, 10th ed. Lippincott Williams & Wilkins.

Borg, G., 1982. Psychophysical bases of perceived exertion. Medicine and Science in Sports and Exercise 14, 377-381. https://doi.org/10.1249/00005768-198205000-00012

Byun, K., Hyodo, K., Suwabe, K., Kujach, S., Kato, M., Soya, H., Ochi, G., Sakairi, Y., Kato, M., Dan, I., Soya, H., 2014a. Possible influences of exercise-intensity-dependent increases in non-cortical hemodynamic variables on NIRS-based neuroimaging analysis during cognitive tasks: Technical note. Journal of Exercise Nutrition & Biochemistry 18, 327-332. https://doi.org/10.1016/j.neuroimage.2014.04.067

Byun, K., Hyodo, K., Suwabe, K., Ochi, G., Sakairi, Y., Kato, M., Dan, I., Soya, H., 2014b. Positive effect of acute mild exercise on executive function via arousal-

related prefrontal activations: an fNIRS study. Neuroimage 98, 336–345. https://doi.org/10.1016/j.neuroimage.2014.04.067

Hanson, N.J., Scheadler, C.M., Lee, T.L., Neuenfeldt, N.C., Michael, T.J., Miller, M.G., 2016. Modality determines VO2max achieved in self-paced exercise tests: validation with the Bruce protocol. European Journal of Applied Physiology 116, 1313–1319. https://doi.org/10.1007/s00421-016-3384-0

Harling, S.A., Tong, R.J., Mickleborough, T.D., 2003. The oxygen uptake response running to exhaustion at peak treadmill speed. Medicine and Science in Sports and Exercise 35, 663–668. https://doi.org/10.1249/01.MSS.0000058434.53664.EC

Harms, C.A., Wetter, T.J., McClaran, S.R., Pegelow, D.F., Nickele, G.A., Nelson, W.B., Hanson, P., Dempsey, J.A., 1998. Effects of respiratory muscle work on cardiac output and its distribution during maximal exercise. Journal of Applied Physiology 85, 609–618. https://doi.org/10.1152/jappl.1998.85.2.609

Joshi, S., Kalwani, R., Gold, J.I., 2016. Relationships between Pupil Diameter and Neuronal Activity in the Locus Coeruleus, Colliculi, and Cingulate Cortex. Neuron 89, 221–234. https://doi.org/10.1016/j.neuron.2015.11.028.Relationships

Kuwamizu, R., Yamazaki, Y., Aoike, N., Hiraga, T., Hata, T., Yassa, M.A., Soya, H., 2023. Pupil dynamics during very light exercise predict benefits to prefrontal cognition. Neuroimage 277, 120244. https://doi.org/10.1016/j.neuroimage.2023.120244

Kuwamizu, R., Yamazaki, Y., Aoike, N., Ochi, G., Suwabe, K., Soya, H., 2022. Pupil-linked arousal with very light exercise : Pattern of pupil dilation during graded exercise. The Journal of Physiological Sciences 72, 1–9. https://doi.org/10.1186/s12576-022-00849-x

McGinley, M.J., David, S.V., McCormick, D.A., 2015. Cortical Membrane Potential Signature of Optimal States for Sensory Signal Detection. Neuron 87, 179–192. https://doi.org/10.1016/j.neuron.2015.05.038

Morgan DW Baldini FD Martin PE, K.W.M., 1989. Ten kilometer performance and predicted velocity at VO2max among well-trained male runners. Medicine & Science in Sports & Exercise 21, 78–83. https://doi.org/10.1249/00005768-198902000-00014

Reimer, J., McGinley, M.J., Liu, Y., Rodenkirch, C., Wang, Q., McCormick, D.A., Tolias, A.S., 2016. Pupil fluctuations track rapid changes in adrenergic and cholinergic activity in cortex. Nat. Commun. 7, 13289. https://doi.org/10.1038/ncomms13289

Richardson, M., Moulton, K., Rabb, D., Kindopp, S., Pishe, T., Yan, C., Akpinar, I., Tsoi, B., Chuck, A., 2016. Capnography for Monitoring End-Tidal CO2 in Hospital and Pre-hospital Settings: A Health Technology Assessment. Capnography for Monitoring End-Tidal CO2 in Hospital and Pre-hospital Settings: A Health Technology Assessment.

Ryu, Y., Maekawa, T., Yoshino, D., Sakitani, N., Takashima, A., Inoue, T., Suzurikawa, J., Toyohara, J., Tago, T., Makuuchi, M., Fujita, N., Sawada, K., Murase, S., Watanave, M., Hirai, H., Sakai, T., Yoshikawa, Y., Ogata, T., Shinohara, M., Nagao, M., Sawada, Y., 2020. Mechanical regulation underlies effects of exercise on serotonin-induced signaling in the prefrontal cortex neurons. iScience 23, 1–16. https://doi.org/10.1016/j.isci.2020.100874

Schwalm, M., Jubal, E.R., 2017. Back to pupillometry: How cortical network state fluctuations tracked by pupil dynamics could explain neural signal variability in human cognitive neuroscience. eNeuro 4. https://doi.org/10.1523/ENEURO.0293-16.2017

Suwabe, K., Byun, K., Hyodo, K., Reagh, Z.M., Roberts, J.M., Matsushita, A., Saotome, K., Ochi, G., Fukuie, T., Suzuki, K., Sankai, Y., Yassa, M.A., Soya, H., 2018. Rapid stimulation of human dentate gyrus function with acute mild exercise. Proc. Natl. Acad. Sci. U. S. A. 115, 10487-10492. https://doi.org/10.1073/pnas.1805668115

Yamada, T., Umeyama, S., Matsuda, K., 2012. Separation of fNIRS signals into functional and systemic components based on differences in hemodynamic modalities. PLoS One 7, e50271. https://doi.org/10.1371/journal.pone.0050271

Yamazaki, Y., Suwabe, K., Nagano-saito, A., Saotome, K., Kuwamizu, R., Hiraga, T., Torma, F., Suzuki, K., Sankai, Y., Yassa, M.A., Soya, H., 2023. A Possible contribution of the locus coeruleus to arousal enhancement with mild exercise : Evidence from pupillometry and neuromelanin imaging. Cerebral Cortex Communications tgad010. https://doi.org/10.1093/texcom/tgad010

Yücel, M.A., Luke, R., Mesquita, R.C., von Lühmann, A., Mehler, D.M.A., Lührs, M., Gemignani, J., Abdalmalak, A., Albrecht, F., de Almeida Ivo, I., Artemenko, C., Ashton, K., Augustynowicz, P., Bajracharya, A., Bannier, E., Barth, B., Bayet, L., Behrendt, J., Khani, H.B., Borot, L., Borrell, J.A., Brigadoi, S., Brink, K., Bulgarelli, C., Caruyer, E., Chen, H.-C., Copeland, C., Corouge, I., Cutini, S., Di Lorenzo, R., Dresler, T., Eggebrecht, A.T., Ehlis, A.-C., Erdoğan, S.B., Evenblij, D., Ferdous, T.R., Fracalossi, V., Franzén, E., Gallagher, A., Gerloff, C., Gervain, J., Goldhamer, N., Gossé, L.K., Guérin, S.M.R., Guevara, E., Hosseini, S.M.H., Innes-Brown, H., Int-Veen, I., Jaffe-Dax, S., Jégou, N., Kawaguchi, H., Kelsey, C., Kent, M., Kessler, R., Kherbawy, N., Klein, F., Kochavi, N., Kolisnyk, M., Koren, Y., Kroczek, A., Kvist, A., Lin, C.-H.P., Löw, A., Luan, S., Mao, D., Martins, G.G., Middell, E., Montero-Hernandez, S., Mutlu, M.C., Novi, S.L., Paquette, N., Paranawithana, I., Parmet, Y., Peelle, J.E., Peng, K., Peng, T., Pereira, J., Pinti, P., Pollonini, L., Jounghani, A.R., Reindl, V., Ringels, W., Schopp, B., Schulte, A., Schulte-Rüther, M., Segel, A., Ala, T.S., Shader, M.J., Shavit, H., Sherafati, A., Soltanlou, M., Sorger, B., Speh, E., Stubbs, K.D., Stute, K., Sullivan, E.F., Tak, S., Tipado, Z., Tremblay, J., Vahidi, H., Van Eeckhoutte, M., Vannasing, P., Vergotte, G., Vincent, M.A., Weiss, E., Yang, D., Yükselen, G., Zapała, D., Zemanek, V., 2025. fNIRS reproducibility varies with data quality, analysis pipelines, and researcher experience. Commun. Biol. 8, 1149. https://doi.org/10.1038/s42003-025-08412-1
